# Molecular noise filtering in the β-adrenergic signaling network by phospholamban pentamers

**DOI:** 10.1016/j.celrep.2021.109448

**Published:** 2021-07-27

**Authors:** Daniel Koch, Alexander Alexandrovich, Florian Funk, Ay Lin Kho, Joachim P. Schmitt, Mathias Gautel

**Affiliations:** 1 Randall Centre for Cell and Molecular Biophysics, King’s College London, SE1 1UL London, UK; 2 Institute of Pharmacology and Clinical Pharmacology, and Cardiovascular Research Institute Düsseldorf (CARID), University Hospital Düsseldorf, 40225 Düsseldorf, Germany

**Keywords:** systems biology, oligomerization, phosphorylation, signaling networks, cardiomyocytes, calcium handling, cardiac arrhythmias, non-linear dynamics, bistability, hysteresis

## Abstract

Phospholamban (PLN) is an important regulator of cardiac calcium handling due to its ability to inhibit the calcium ATPase SERCA. β-Adrenergic stimulation reverses SERCA inhibition via PLN phosphorylation and facilitates fast calcium reuptake. PLN also forms pentamers whose physiological significance has remained elusive. Using mathematical modeling combined with biochemical and cell biological experiments, we show that pentamers regulate both the dynamics and steady-state levels of monomer phosphorylation. Substrate competition by pentamers and a feed-forward loop involving inhibitor-1 can delay monomer phosphorylation by protein kinase A (PKA), whereas cooperative pentamer dephosphorylation enables bistable PLN steady-state phosphorylation. Simulations show that phosphorylation delay and bistability act as complementary filters that reduce the effect of random fluctuations in PKA activity, thereby ensuring consistent monomer phosphorylation and SERCA activity despite noisy upstream signals. Preliminary analyses suggest that the PLN mutation R14del could impair noise filtering, offering a new perspective on how this mutation causes cardiac arrhythmias.

## Highlights

• A dynamical systems model of the PLN signaling network is developed
• PLN pentamers enable low-pass filtering (LPF) by competing with PLN monomers
• PLN pentamers promote the emergence of bistable PLN phosphorylation
• LPF and bistability filter out simulated signal noise in the β-adrenergic pathway

## Graphical abstract

**Figure:**
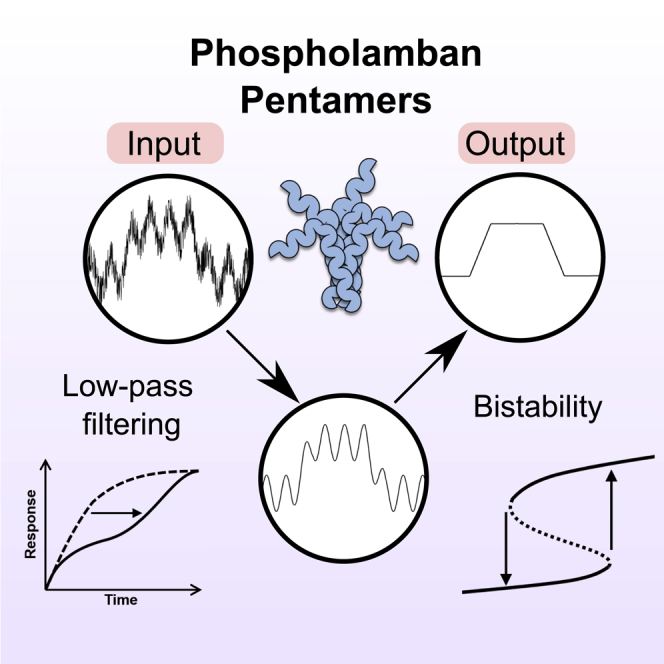

## Teaser

While phosphorylation of phospholamban (PLN) is crucial to improve heart function during the β-adrenergic “fight-or-flight” response, the role of PLN pentamers in this context is unclear. By integrating mathematical and experimental approaches, Koch et al. find that PLN pentamers provide complex information-processing capabilities to ensure a consistent phosphorylation response.

## Introduction

Calcium (Ca^2+^) currents determine contraction and relaxation of the heart at the cellular level: high Ca^2+^ concentrations enable sarcomeric contraction, whereas low Ca^2+^ concentrations lead to relaxation (Bers, 2002; Eisner et al., 2017). These currents are controlled by the release and reuptake of calcium from and into the sarcoplasmic reticulum (SR), the major storage compartment for intracellular Ca^2+^. At the molecular level, dozens of proteins regulate Ca^2+^-handling and excitation-contraction coupling (Bers, 2008). The Ca^2+^ pump sarco/endoplasmic reticulum Ca^2+^-ATPase (SERCA) mediates ∼70%–90% of the Ca^2+^ reuptake into the SR and therefore induces relaxation of the cardiomyocyte (Bers, 2002; MacLennan and Kranias, 2003). SERCA function is inhibited by phospholamban (PLN), a 52-amino acid protein resident in the SR membrane. Phosphorylation of PLN at Ser16 by protein kinase A (PKA) reverses SERCA inhibition in response to β-adrenergic stimulation, thereby accelerating Ca^2+^ removal and cardiomyocyte relaxation (Tada et al., 1975; Kranias and Solaro, 1982; Lindemann et al., 1983; MacLennan and Kranias, 2003; Kranias and Hajjar, 2012). This constitutes an important mechanism to adapt cardiac output to increasing demand and is an integral part of the β-adrenergic “fight-or-flight” response (Simmerman and Jones, 1998; MacLennan and Kranias, 2003; Kranias and Hajjar, 2012). Disruptions in this part of the β-adrenergic signaling network can have drastic consequences. Multiple mutations in the PLN gene have been discovered in the past two decades, most of which cause severe forms of cardiomyopathy and lead to cardiac arrhythmias or heart failure (Schmitt et al., 2003, 2009; Haghighi et al., 2006; Medeiros et al., 2011; Yost et al., 2019).

In spite of the progress in understanding the structure and function of PLN, many aspects of this protein are still poorly understood and specific therapeutic approaches to manipulate the PLN signaling network are lacking. One of the less-well-understood aspects is the assembly of PLN into homo-pentamers (Wegener and Jones 1984). Although their pinwheel-like structure in lipid environments (Verardi et al., 2011) yields intuitive plausibility to early conjectures and data suggesting that pentameric PLN acts as an ion channel (Kovacs et al., 1988; Smeazzetto et al., 2016), this hypothesis has been contested by multiple experimental, structural, and theoretical studies (Maffeo and Aksimentiev, 2009; Becucci et al., 2009; Vostrikov et al., 2013). Since an artificial monomeric PLN mutant was found to be a similarly potent SERCA inhibitor as wild-type PLN (Kimura et al., 1997), the prevailing paradigm considers pentamers to be a biologically inactive storage form (MacLennan and Kranias, 2003; Becucci et al., 2009; Kranias and Hajjar, 2012). Increasing evidence suggests, however, that PLN pentamers are not entirely passive and influence cardiomyocyte contractility and PLN phosphorylation dynamics (Colyer, 1993; Chu et al., 1998; Wittmann et al., 2015; Glaves et al., 2019). Vostrikov et al. (2013) proposed that pentamers could act as buffers that fine-tune SERCA regulation via monomeric PLN by keeping it within a physiological window. However, it is not obvious exactly what benefit pentamerization contributes, given that SERCA activity can already be controlled by regulating expression levels and by multiple post-translational modifications of both PLN and SERCA (McTiernan et al., 1999; Stammers et al., 2015; MacLennan and Kranias, 2003). The specific physiological advantage of pentamerization and its role in the pathophysiology of PLN mutations thus remains elusive.

In the present study, we investigated the role of pentamers in the PLN regulatory network. We found that pentamers have only a limited capacity to buffer the concentration of monomeric PLN *in vitro* since the effect is slow and moderate. Based on the hypothesis that the function of pentameric PLN exceeds monomer buffering, we developed a mathematical model of the PLN regulatory network to study the role of pentamers in the context of β-adrenergic stimulation from a dynamical systems perspective. Our results indicate that pentamers are molecular noise filters to ensure consistent PLN phosphorylation in response to noisy β-adrenergic stimulation. A preliminary analysis of the arrhythmogenic PLN mutation R14del suggests that this mutation could impair noise filtering, indicating that molecular noise filtering in the β-adrenergic signaling network could be important to prevent cardiac arrhythmias.

## Results

### Pentamers are moderate and slow monomer buffers *in vitro*

**Figure 1:**
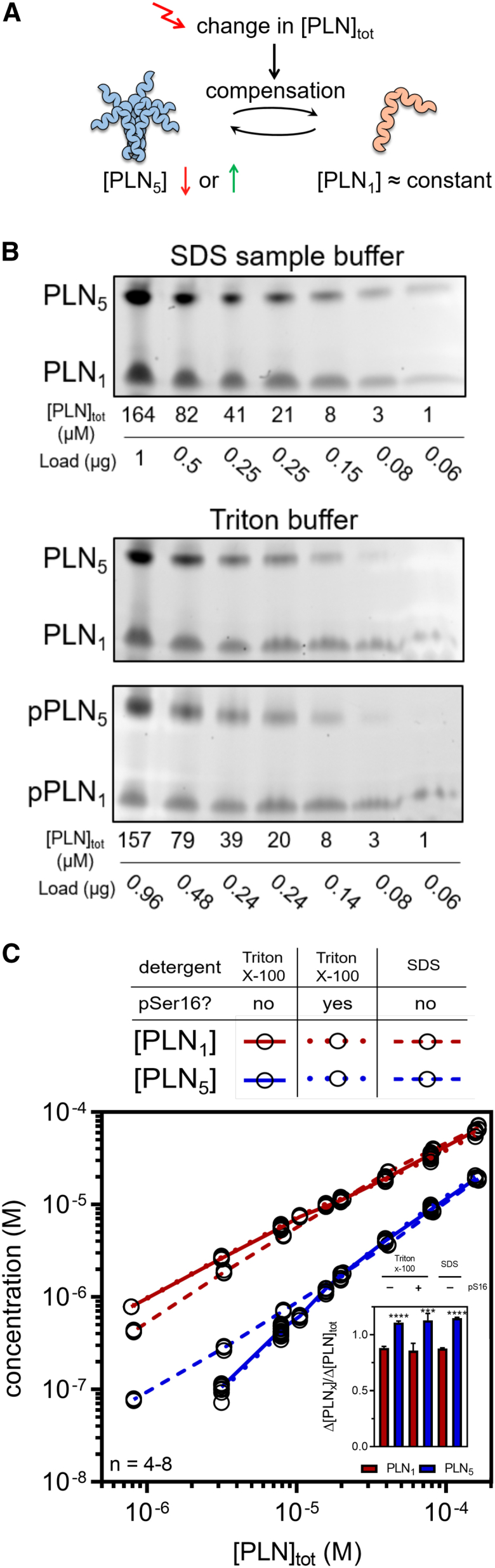
*In vitro* oligomerization (A) In the prevailing paradigm, pentamers buffer the PLN monomer concentration by compensating changes via association or dissociation. (B and C) Oligomeric state of PLN in SDS sample or Triton X-100-based buffer (TBB) at different total protein concentrations after dilution. (B) Oriole-stained gels after semi-native SDS-PAGE. (C) Quantified monomers and pentamers at different total concentrations. Inset: relative change in monomers and pentamers when comparing the highest 2 total concentrations. ^∗∗∗^p < 0.001,^∗∗∗∗^p < 0.0001 versus monomers.

The predominant paradigm is that PLN pentamers are a storage or buffering reservoir for monomers (Figure 1A) (MacLennan and Kranias, 2003; Becucci et al., 2009; Kranias and Hajjar, 2012; Vostrikov et al., 2013). The oligomeric state of PLN in tissue or cell homogenates is typically assessed from samples in SDS sample buffer, which does not interfere with oligomerization. However, SDS is a harsh anionic detergent that interferes with the function of many other proteins. We therefore studied PLN oligomerization in both SDS sample buffer and a Triton X-100 based buffer (TBB) at physiological pH and ionic strength, which effectively solubilizes PLN and allows for rapid phosphorylation of PLN at Ser16 by PKA.

To test the hypothesis that PLN pentamers buffer monomer concentration, we analyzed the oligomeric state of PLN (unphosphorylated and phosphorylated) by semi-native SDS-PAGE at various total PLN concentrations after dilution and 2 h of equilibration (Figure 1B). As shown in Figure 1C, the slope of pentameric PLN in TBB is steeper than the slope of monomeric PLN (particularly at low total concentrations), suggesting that changes in total PLN concentration have a larger effect on pentameric than on monomeric PLN. In contrast to the experiments in TBB, pentameric PLN appears not to dissociate upon dilution in SDS sample buffer. In the likely region of physiological PLN concentrations at the SR (>50 μM; Star Methods table: review protein concentrations), the change in monomers relative to the change in total PLN is slightly lower than for pentamers, indicating that pentamers can buffer the concentration of monomers (Figure 1C, inset). However, we found that pentamers dissociate only slowly, with an apparent mean lifetime ${\left(k_{obs}\right)}^{-1}$ of 11.4 min for pentamers (Figure S1A), in good agreement with previous live-cell measurements (Robia et al., 2007). We concluded that under the investigated *in vitro* conditions, PLN pentamers buffer the concentration of PLN monomers only moderately and slowly. We thus hypothesized that PLN pentamers may play further roles.

On a site note, we observed no increased pentamerization upon phosphorylation of PLN in TBB (Figures 1B, 1C, and S1B). The increase in pentamerization upon phosphorylation is sometimes called the dynamic equilibrium of PLN and while its biological significance is unclear, it has been speculated that it might contribute to SERCA regulation (Cornea et al., 1997; Hou et al., 2008; Kranias and Hajjar, 2012). Interestingly, we observed a significant increase in pentamerization after diluting PLN phosphorylated in TBB with SDS sample buffer (Figure S8E), indicating that the effect relies on anionic environments.

### A mathematical model of the PLN regulatory network

Mathematical modeling has been paramount to understand the non-linear behavior of signaling networks and how they regulate cellular activities including growth, differentiation, apoptosis, and motility. PLN is also part of a complex signaling network involving multiple kinases, phosphatases, and regulatory complexes, a network that so far remained largely unexplored by mathematical approaches. Although a PLN submodule is part of several models of cardiac Ca^2+^ cycling (Bugenhagen and Beard, 2015) or β-adrenergic signal transduction (Saucerman et al., 2003), no mathematical model has, to our knowledge, considered PLN pentamers or provided a detailed analysis of the network immediately implicated in regulating PLN. Aiming to fill this gap, we set out to develop a mathematical model of the PLN network to study its functionality and the role of pentamers in the context of β-adrenergic stimulation from a dynamical systems perspective.

We began model development by considering several possible models of how PLN forms pentamers and calibrated them using our dilution and dissociation time course data. We found that a model following a monomer→dimer→tetramer→pentamer pathway shows good agreement with our experimental data and outperforms other model variants (see STAR Methods section “Development of the mathematical model” and Figures S9 and S10). We extended the PLN oligomerization model by including key proteins and reactions of the β-adrenergic signal transduction network involved in regulating the phosphorylation of PLN at Ser16. We accounted for reactions and enzymes responsible for addition (PKA) and removal of the Ser16 phosphate group (phosphatases PP1 and PP2A) (MacDougall et al., 1991; MacLennan and Kranias, 2003). Dephosphorylation of PLN pentamers has been shown to exhibit strong positive cooperativity (Li et al., 1990). Since PP1 is the main phosphatase for reversing Ser16 phosphorylation of PLN (MacDougall et al., 1991; Steenaart et al., 1992), we assumed that the catalytic turnover for dephosphorylation of pentameric PLN by PP1 increases with fewer phosphate groups left on a pentamer. We implemented this assumption by introducing dimensionless parameters φ and χ for tuning individual steps of pentamer dephosphorylation by PP1 (Figure S12C). We also included the regulation of PP1 by inhibitor-1 as described in Saucerman et al. (2003). Inhibitor-1 can bind and inhibit PP1 when phosphorylated by PKA at Thr35, whereas phosphorylation at this site is reversed by PP2A (Kranias and Hajjar, 2012; Saucerman et al., 2003). To keep our analysis focused on the regulation of PLN phosphorylation in the context of β-adrenergic stimulation, we treated the concentration of active PKA at the SR as a model input parameter and omitted processes upstream of PKA (e.g., cAMP production and degradation) and downstream of PLN (e.g., SERCA activity, Ca^2+^handling). Due to the lack of mechanistic and kinetic data, we did not include (de-)phosphorylation of PLN at Ser10 or Thr17.

**Figure 2:**
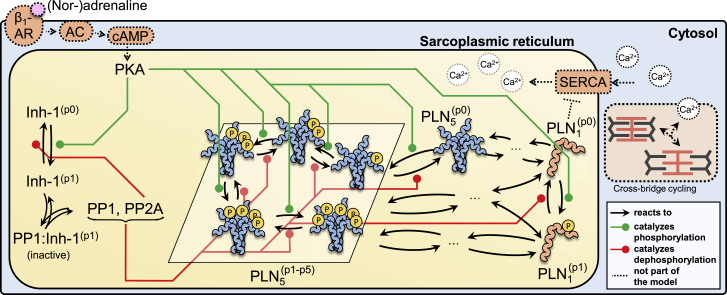
Simplified model scheme The depicted signaling network controls SERCA activity in response to β-adrenergic stimulation via regulation of PLN phosphorylation. The model captures all of the processes immediately involved in regulating PLN phosphorylation at Ser16. To simplify the scheme, the different oligomerization routes of phosphorylated and unphosphorylated PLN are not shown. See Method details for model equations and parameter values and Figure S12A for the complete model scheme.

Figure 2 shows a simplified scheme of the biochemical reactions included in our model. The model comprises 60 biochemical reactions between 20 molecular species that are described by a set of 17 ordinary differential and 3 algebraic equations. The additional protein concentrations and model parameters not determined by our own data are based on experimental measurements from the literature.

A more detailed description of how the model was formulated can be found in the Method details section along with the model equations and parameter values. Having a mathematical description of the processes that regulate PLN phosphorylation at our disposal, we set out to explore the behavior of our model.

### Pentamers and the inhibitor-1 feedforward loop delay monomer phosphorylation

**Figure 3:**
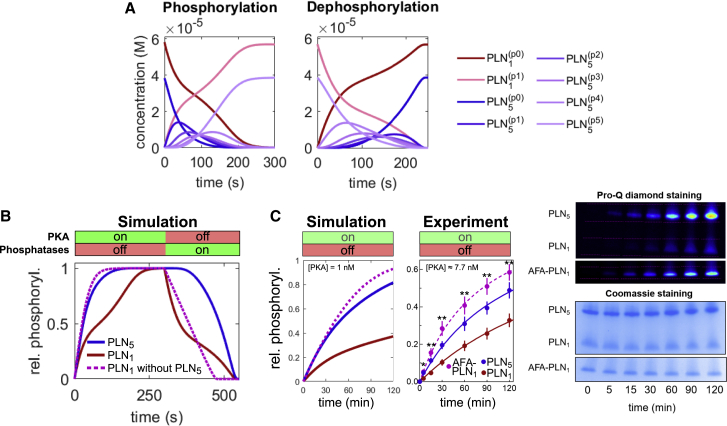
Regulation of phosphorylation dynamics by PLN pentamers (A) Time course simulations of PLN phosphorylation by 0.1 μM PKA in the absence of phosphatases (left) and dephosphorylation of completely phosphorylated PLN by PP1 and PP2A in the absence of PKA (right). (B) Dynamics of relative phosphorylation levels in a sequential phosphorylation/dephosphorylation simulation in the presence or absence of pentameric PLN. (C) Simulated and experimental phosphorylation time course of wild-type PLN ([PLN_*tot*_] ≈ 157 μM, at which [PLN_1_] ≈ 52 μM) and monomeric AFA-PLN_1_ (≈52 μM). A low PKA concentration was chosen to slow down the reaction for easier sampling. Data represent means ± SEMs. ^∗^p < 0.05, ^∗∗^p < 0.01, AFA-PLN_1_ versus PLN_1_.

In a first simulation, we studied the dynamics of PLN monomer and pentamer phosphorylation by PKA in the absence of phosphatases (Figure 3A, left). The phosphorylation of monomers resembles a hyperbola but features a kink in the middle. Pentamer phosphorylation, however, exhibits dynamics typical for multisite phosphorylation systems with transient waves of incompletely phosphorylated intermediate forms. Next, we simulated the dephosphorylation of completely phosphorylated PLN in the absence of PKA (Figure 3A, right). As expected, dephosphorylation resembles the phosphorylation dynamics but in reverse order. As expected from the implemented cooperativity of PP1, the accumulation of unphosphorylated pentamers is more abrupt.

To simplify the plots, we decided to focus on relative PLN phosphorylation for the remainder of this study. Interestingly, re-plotting the data from the phosphorylation time course simulations reveals that relative phosphorylation of monomers significantly lags behind the relative phosphorylation of pentamers (Figure 3B). A likely explanation for this delay could be that monomers and pentamers compete against one another as PKA substrates. Performing the same simulation without pentamers but at an equimolar monomer concentration abolishes delayed phosphorylation, confirming that the lag is caused by competing PLN pentamers (Figure 3B, dotted line). Parameters that increase substrate competition or, surprisingly, slow down pentamer phosphorylation, can increase this delay (Figure S2A).

To test the predicted delay experimentally, we carried out PKA-phosphorylation time course experiments using wild-type PLN and AFA-PLN (an artificial monomeric mutant) at equimolar monomer concentrations. In agreement with the simulations, we found monomer phosphorylation to be significantly delayed in the presence of pentamers (Figure 3C).

**Figure 4:**
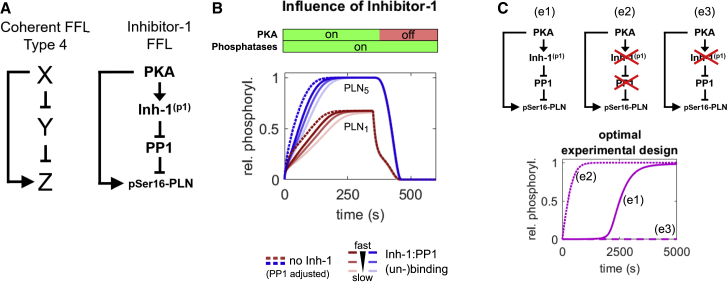
Regulation of phosphorylation dynamics by inhibitor-1 (A) Left: coherent feed-forward loop (FFL) type 4 in which the full response of Z is delayed until the inhibitory effect of Y is revoked by X. Right: structure of the inhibitor-1 FFL. (B) Influence of the inhibitor-1 FFL in simulations of PLN phosphorylation by PKA in the presence of PP1. To ensure equal steady-state phosphatase activity in the absence of inhibitor-1, PP1 levels have been adjusted by the amount in complex with inhibitor-1 in presence of PKA at steady state in the full model. (C) Optimal experimental design and controls for detecting PLN phosphorylation delay by the inhibitor-1 FFL is given by [PKA] < [PP1] < [Inh-1] and indicated knockout versions of the FFL.

Substrate competition is not the only network motif able to delay the response to a stimulus. Interestingly, the PLN network contains a second motif with such ability: the inhibition of PP1 by PKA via the phosphorylation of inhibitor-1 constitutes a subgraph that can be described as an elongated version of a coherent type 4 feed-forward loop (FFL) able to cause delays (Figure 4A) (Mangan and Alon, 2003). Simulations show that inhibitor-1 can delay the phosphorylation of PLN monomers and pentamers if the binding of phosphorylated inhibitor-1 to PP1 is not too fast (Figure 4B). Reducing the PP1 concentration by the fraction that is inhibited by inhibitor-1 at steady state in the presence of PKA and repeating the simulation in the absence of inhibitor-1 shows that PLN phosphorylation approaches the same steady-state levels, but much faster (Figure 4B, dotted lines). For slower inhibitor-1 phosphorylation, the delay becomes more pronounced (Figure S2B). The delay can be uncoupled from pentamer competition by using monomeric AFA-PLN and maximized when [PKA] < [PP1] < [Inh-1]. When contrasted to knockout variants of the FFL, this yields an optimal design for future experimental testing of the predicted delay (Figure 4C).

In summary, our simulations predict the existence of two independent response delay elements in the PLN network: pentamers delaying the phosphorylation of monomers and an inhibitor-1 FFL delaying the phosphorylation of both monomers and pentamers.

### Bistability in the steady-state phosphorylation of PLN

Mangan and Alon (2003) proposed that response delay elements may act as persistence sensors that reject short input stimuli. Before exploring what the physiological advantage of such persistence sensing in the context of β-adrenergic stimulation may be, we shall first consider how PLN phosphorylation is controlled at steady state.

**Figure 5:**
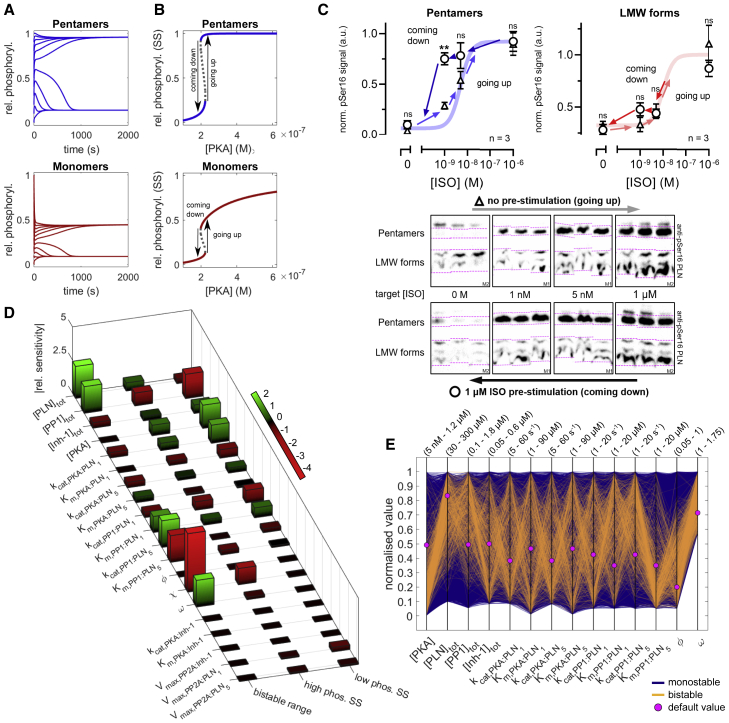
PLN phosphorylation at steady state (A) Time course simulations with different initial levels of PLN phosphorylation show hysteresis ([PKA] = 0.21 μM). (B) Bifurcation diagrams for relative phosphorylation of PLN monomers and pentamers show a bistable region of ≈0.025 μM range. (C) Western blot analysis of PKA-dependent steady-state PLN phosphorylation confirms the predicted hysteresis in neonatal rat cardiomyocytes. Pre-stimulated cardiomyocytes were treated for 2 min with 1 μM isoprotenerol before the concentration was lowered to the target isoprotenerol concentration. Bands shown in logical order; only signals from the same membrane were compared (M1/M2 in bottom corner). Arrows indicate the direction of hysteresis loop. Data represent means ± SEMs. ^∗∗^p < 0.01 pre-stimulated versus non-pre-stimulated. (D) Local sensitivity analysis of low phosphorylation steady state ([PKA] = 0.13 μM), high phosphorylation steady state ([PKA] = 0.25 μM), and the range of the bistable region. Relative sensitivities determined at Δ*p* = +1% for high/low phosphorylation steady states and Δ*p* = +10% for bistable range. (E) Parallel coordinate plot of the model stability behavior for 10,000 random parameter sets.

Multisite phosphorylation systems can exhibit ultrasensitivity and bistability if there is sufficient kinetic asymmetry in the subsequent cycles of phosphorylation and dephosphorylation (e.g., due to cooperativity or multi-enzyme regulation) (Koch, 2020; Markevich et al., 2004). Since cooperativity is present in the dephosphorylation of pentameric PLN (Li et al., 1990), we wondered whether PLN phosphorylation may be bistable at some level of PKA activity. A hallmark of bistability is that the approached steady state depends on the system’s history (hysteresis). We therefore performed several simulations with identical settings except for different initial levels of relative phosphorylation and found that PLN phosphorylation is indeed bistable at some PKA concentrations (Figure 5A). To better understand the steady-state phosphorylation of PLN, we generated bifurcation diagrams, which visualize how this non-linear system behavior depends on PKA concentration as a control parameter. We found that PLN phosphorylation increases in an abrupt, ultrasensitive fashion as it passes a threshold at approximately one-third of the maximum PKA concentration at the SR (≈0.6 μM; Saucerman et al., 2003; Figure 5B).

Since ultrasensitivity is considered a prerequisite and indication for bistability, we re-analyzed previous experimental dose-response data by fitting it to the Hill equation and found Hill exponents of ≈2 (Figure S3A). The data from Wittmann et al. (2015) suggest that this ultrasensitivity depends on the presence of pentamers (Figure S3A), likely due to the (pseudo-)multisite nature of pentamers (Koch, 2020). We were further able to replicate the ultrasensitive dose-response of PLN Ser16 phosphorylation in transfected HEK293 cells after PKA activation by forskolin (Figure S3B). Despite the experimental uncertainty in the value of the Hill exponents, the data together indicate some degree of ultrasensitivity in PLN phosphorylation. Since these data are based on the average response of the network across cells, it is possible that the Hill exponent at the single-cell level is even higher (Figure S3C).

Next, we sought to determine experimentally whether PLN phosphorylation in cardiomyocytes exhibits hysteresis as predicted by our model. This can be achieved by comparing the steady-state phosphorylation of pre-stimulated/non-pre-stimulated cells. Due to our simulations, we expected hysteresis to most likely occur slightly below but close to the region of highest dose sensitivity. Based on previous dose-response data (EC_50_ ≈ 5 nM isoprotenorol; Kuschel et al., 1999), we chose to test for hysteresis in the steady-state phosphorylation of PLN at isoprotenorol concentrations of 1 and 5 nM by western blotting. As shown in Figure 5C, we found pronounced hysteresis for pentamer steady-state phosphorylation at 1 nM isoprotenorol. In contrast, no hysteresis was observed at 0 or 1 μM isoprotenorol, demonstrating that hysteresis is specific to a region of criticality. Unfortunately, monomer bands were poorly separated from other small oligomeric forms and thus were summarized as low-molecular-weight (LMW) forms, but a significant difference could not be observed. Taken together, our experimental results demonstrate that PLN phosphorylation is ultrasensitive and bistable.

To find out which model parameters exert the most control over PLN phosphorylation at steady state, we performed a local sensitivity analysis of relative monomer phosphorylation (Figure 5D). We found that the low phosphorylation state is generally more sensitive to parameter perturbations than the high phosphorylation state, but both are primarily controlled by concentrations and catalytic constants of PKA and PP1. Parameters associated with inhibitor-1 or PP2A show only a minor influence. The bistable range depends primarily on parameters that influence PP1 cooperativity or substrate competition between PLN monomers and pentamers (e.g., higher concentrations of PLN and PP1 or changes to PP1 dependent dephosphorylation). Interestingly, the cooperative increase in substrate affinity (χ) and the dynamic equilibrium of PLN (ω) show a strong influence on the bistable range. In the default parameter set, we assume PLN turnover $\left(k_{cat}\right)$ rather than substrate affinity $\left(K_{m}\right)$ to be regulated cooperatively (i.e., χ = 1 and φ < 1), but the exact nature of cooperative PLN pentamer dephosphorylation is currently unknown. While cooperative increase of $k_{cat}$ is essential for the emergence of bistability, increasing substrate affinity appears to reduce the bistable range. To better understand how parameters φ, χ, and ω shape the PLN phosphorylation response curve, we performed further bifurcation analyses. Although low φ and χ or high ω values can increase the bistable range, the parameters differ markedly in how they shape other characteristics of the dose-response curve, possibly due to distinct effects on dephosphorylation rates (Figures S4A and S4B).

Local sensitivity analysis permits the study of the influence of parameters only around a nominal steady state, limiting the generality of its conclusions, whereas bifurcation analysis can be challenging and is limited to varying only few parameters simultaneously. We therefore implemented a recently developed method that allows exploring models in a fashion unbiased by a particular parameter set by simultaneously probing an arbitrary subset of the multi-dimensional parameter space and visualizing the resulting stability behavior on parallel coordinate plots (Nguyen et al., 2015). For each analysis, we probed 10,000 randomly sampled parameter sets, focusing on the concentrations of PKA, PP1, PLN, and inhibitor-1, enzymatic constants, cooperativity parameter φ, and the dynamic equilibrium of PLN (ω) (Figure 5E). In the absence of cooperative substrate affinity of PLN dephosphorylation (χ = 1), 5.5% of the sampled parameter sets led to bistable phosphorylation responses. The emergence of bistability is favored by high $k_{cat}$ and low $K_{m}$ values for pentamer dephosphorylation by PP1. In contrast, other PKA and PP1 constants exhibit relatively little influence. Bistability is furthermore associated with low [PKA], high [PLN]_*tot*_, and [PP1]_*tot*_ as well as a strong cooperative increase in $k_{cat}$ of PP1 (low φ values) and strong dynamic equilibrium (high ω values). To further study the role of pentameric PLN and the nature of PP1 cooperativity in pentamer dephosphorylation, we repeated the analysis without pentameric PLN and with cooperative substrate affinity of PLN dephosphorylation (χ > 1), respectively. We found no bistability in the absence of pentameric PLN and markedly fewer (1.1%) parameter sets leading to bistability when χ > 1 (Figures S4C and S4D).

In summary, these analyses show that pentamers, their cooperative dephosphorylation, and the dynamic equilibrium of PLN are important factors in shaping PLN monomer phosphorylation response at steady state.

### Phosphorylation delay and bistability are effective noise filters

Like the phosphorylation response delay, the emergence of bistability poses the question what the physiological advantage of such phenomenon may be. Due to the small bistable range, it seems unlikely that PLN phosphorylation is a potent all-or-nothing switch as known for bistable signaling networks controlling (e.g., the cell cycle, apoptosis). In fact, adapting cardiac performance to various levels of demand requires the response to β-adrenergic stimulation to be tunable.

Altered Ca^2+^ handling is a known cause for cardiac arrhythmias (Landstrom et al., 2017). Cardiac arrhythmias such as ventricular tachycardias and fibrillation are also a hallmark of the pathogenic PLN mutation R14del (Haghighi et al., 2006; Posch et al., 2009; van Rijsingen et al., 2012; Hof et al., 2019). We thus speculated that delayed and bistable PLN phosphorylation may play a role in preventing such arrhythmias. If the phosphorylation delay is a persistence sensor (Mangan and Alon, 2003) for β-adrenergic stimulation, then it indicates that the “decision” of a cardiomyocyte to phosphorylate PLN may be a critical one. We hypothesized that by controlling PLN phosphorylation, response delay and bistability are noise-filtering mechanisms to prevent random, uncoordinated β-adrenergic signaling and aberrant Ca^2+^ handling.

**Figure 6:**
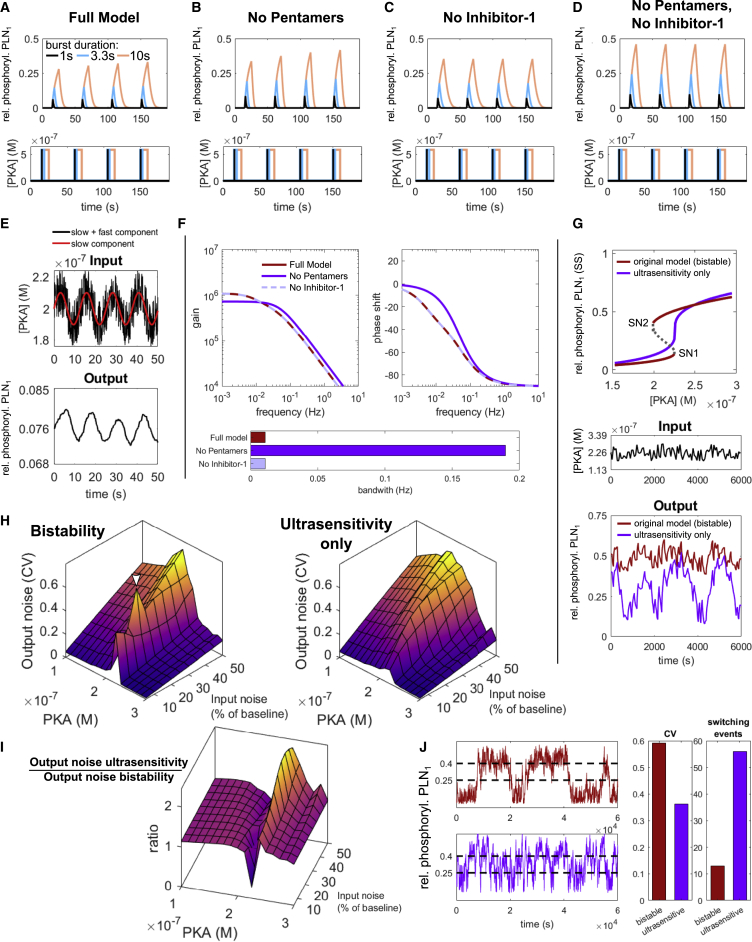
Noise filtering by the PLN network (A–D) Time course simulations of PLN monomer phosphorylation in response to short (1/3.3/10 s) bursts of maximal PKA activity performed with the full model (A) or in the absence of pentameric PLN and/or inhibitor-1 (B–D). To ensure equal steady-state phosphatase activity, PP1 levels in (C) and (D) have been adjusted by the amount in complex with inhibitor-1 in presence of PKA at steady state in the full model. (E) Demonstration of the PLN network’s low pass filtering capacity. (F) Frequency response analysis (Bode plots) of the linearized input-output systems. (G) Comparison of PLN monomer phosphorylation (bottom) in response to a noisy PKA input fluctuating with 25% min^−1^ around a baseline of 0.226 μM (center) for the original model and a model with similar steady-state response but without bistability (top). (H) Output noise as the coefficient of variation *σ/μ* of monomer phosphorylation for the original (bistable) and ultrasensitive model at different PKA baseline and input noise levels. (I) Output noise of the ultrasensitive model relative to the bistable model. (J) Comparison of bistable and ultrasensitive model at critical PKA concentrations and a maximum noise amplitude (0.0625 μM), which enables repeated switching between low/high phosphorylation (dashed lines) in both models.

To test this hypothesis, we performed a series of different simulations and analyses to characterize the noise-handling behavior of the model in response to random fluctuations of PKA activity. In the first simulations, we explored monomer phosphorylation in response to short bursts (1/3.3/10 s) of maximal PKA activity (0.59 μM) in the full model, in the absence of either pentamers or inhibitor-1, and in the absence of both pentamers and inhibitor-1 (PP1 levels were adjusted to ensure equal steady-state activity in the absence of inhibitor-1). In the full model, the first 1/3.3/10 s bursts lead to 6%/13%/28% monomer phosphorylation, respectively (Figure 6A). In the absence of either pentamers or inhibitor-1, the response to such bursts is markedly higher, reaching 10%/23%/46% for 1/3.3/10 s bursts in the absence of both pentamers and inhibitor-1 (Figures 6B–6D). As expected from the response delays, a comparison of the integrated monomer phosphorylation between model versions reveals that relative attenuation is strongest for short bursts (Figure S5A). Interestingly, when inhibitor-1 is present, unbinding and subsequent dephosphorylation of phosphorylated inhibitor-1 occurs more slowly than the dephosphorylation of PLN, leading to the accumulation of the inactive PP1 complex and slightly increasing PLN phosphorylation over multiple bursts (Figures 6A and 6B). These simulations show that the response delay via pentamers and inhibitor-1 can filter out or attenuate short PKA activity bursts while still allowing high phosphorylation upon persistent PKA activity.

Rejecting signals on short timescales while responding to persistent signals is also characteristic of low-pass filters. Simulating the PLN phosphorylation response to a PKA input described by a low-frequency sine wave interspersed with high-frequency random noise confirms that the PLN signaling network has low-pass filtering properties (Figure 6E). Such behavior can be further characterized by a frequency response analysis that permits the determination of the bandwidth (i.e., the frequency above which a system fails to response adequately). Typical for low-pass filters, the gain Bode plot of our model shows a steady decrease in the gain (roll-off) for frequencies above the bandwidth (Figure 6F). Consistent with our previous analysis, the bandwidth is 17-fold higher in the absence of pentamers (0.196 Hz) compared to the full model (0.011 Hz) (Figure 6F, bar graph), confirming that pentamers contribute to low-pass filtering in the PLN network. Interestingly, the phase shift is also different in the absence of pentamers, further substantiating their role in influencing phosphorylation dynamics.

To our surprise, the absence of inhibitor-1 did not increase the bandwidth (contrary to what would be expected from the demonstrated response delay). The reason for this is that the frequency response is constructed from the reached steady state. Due to the high affinity of inhibitor-1 for PP1, 99.8% of inhibitor-1 at the studied steady state in the full model is already bound to PP1 and does not contribute to low-pass filtering anymore. Unless inhibitor-1 can be dephosphorylated by PP2A while bound to PP1 (which to our knowledge has not been studied yet), its response delay would only apply if the cardiomyocyte has not been exposed to significant β-adrenergic stimulation for some time.

Our simulations and analyses show that the response delay by PLN pentamers and inhibitor-1 can attenuate the response to short bursts of PKA activity and that at least pentamers contribute to low-pass filtering. Next, we explored how bistability may contribute to noise filtering. In general, bistability can make a response more robust and defined: once a system passed a threshold, it can only switch back to its prior state if it passes a second threshold, thus preventing uncontrolled switching (Ferrell and Xiong, 2001). We thus speculated that bistability could reduce noise by preventing repeated switching between low/high PLN phosphorylation levels. To test this hypothesis, we created a parameter set for which the model shows similar monomer phosphorylation at steady state in terms of sensitivity and critical threshold but without bistability (Figure 6G, top).

Next, we compared the behavior of both parameterizations in response to noisy PKA activity close to the common critical threshold. Fluctuations of 25% with a frequency of 1 min^−1^ have been chosen to make sure the fluctuations are not filtered out by low-pass filtering (Figure 6G, center). As shown in Figure 6G (bottom), the relative PLN monomer phosphorylation of the bistable model (red) fluctuates with small amplitude around a stable baseline of ∼50% phosphorylation. In contrast, the non-bistable model (purple) shows dramatic fluctuations between low and high phosphorylation levels. Since PLN monomer phosphorylation directly translates into SERCA activity, such fluctuations could impair coordinated Ca^2+^-handling.

To investigate the output noise in a more systematic manner, we applied a common definition of signal noise as the coefficient of variation (CV) (Johnston, 2012). By calculating “noise landscapes” for both models based on 150 PKA fluctuations with a frequency of 1 min^−1^, we visualized how the CV of monomer phosphorylation (output noise) depends both on the baseline PKA activity and amplitude of PKA fluctuations (input noise). While the output noise of bistable and non-bistable model versions is very similar for baseline [PKA] below ≈0.2 μM, the output noise of the bistable model abruptly increases at a baseline [PKA] close to the critical threshold and abruptly decreases at higher [PKA] (Figure 6H, left). The output noise of the non-bistable model follows a more continuous trend and neither shows abrupt increases close to the critical threshold, nor abrupt suppression at higher baseline [PKA] (Figure 6H, right). The relative noise landscape shows that in most circumstances, the bistable model copes better with noisy input than the non-bistable model (Figure 6I). Since we assumed the input noise to be a linear function of the baseline [PKA], we repeated the analyses assuming a constant and a non-linear noise function and came to the same conclusion (Figures S5B–S5D).

Since the bistable model seemingly performs worse in some conditions close to the critical threshold, the question arises whether this could facilitate cardiac arrhythmias in spite of a generally less noisy monomer phosphorylation. To answer this question, we analyzed one of the conditions in which the bistable model seemingly performs worse (white arrowhead in Figure 6H) in more detail. Interestingly, we found that the increased output noise as defined by the CV typically resulted from a single “switching up” event and that in the long run (1,000 fluctuations), the output noise of the bistable model is actually lower than in its non-bistable counterpart (Figure S5E).

Motivated by this finding, we wanted to know how bistable and non-bistable model versions compare at their most vulnerable point for uncontrolled switching between low/high monomer phosphorylation states. We thus designed simulations in which baseline [PKA] was set to the center between both saddle-node bifurcations for the bistable model (i.e., between critical thresholds SN1 and SN2 shown in Figure 6G) or directly to the single threshold in the non-bistable model. In addition, we chose a constant maximum noise amplitude for both models, high enough to surpass both thresholds in the bistable model from its baseline [PKA]. Intriguingly, we found that in spite of a higher CV, monomer phosphorylation is more defined and switches far less frequently in the presence of bistability (Figure 6J).

In summary, these simulations and analyses confirm our hypothesis that phosphorylation delay and bistability can act as molecular noise filters in the β-adrenergic signaling network.

### The R14del mutation likely impairs noise filtering

Coordinated functioning of the heart critically depends on the synchronicity of cardiomyocyte contraction and relaxation controlled by intracellular [Ca^2+^]. Since β-adrenergic stimulation is a major regulator of cardiac Ca^2+^ handling, there likely need to be mechanisms in place to prevent arrhythmias triggered by heterogeneous cardiomyocyte responses. By rejecting short random stimuli (low-pass filtering) and by defining the PLN phosphorylation status more clearly (bistability), these noise filters could help to promote synchronicity across the myocardium.

Since cardiac arrhythmias are a major issue for patients with the PLN mutation R14del (Haghighi et al., 2006; Posch et al., 2009; van Rijsingen et al., 2012; Hof et al., 2019), we wanted to know whether noise filtering is impaired if we implement the known molecular effects of this mutation into our model. The consequences reported so far include impaired phosphorylation by PKA (Kim et al., 2015) (although Haghighi et al. [2006, 2012] reported that PLN_*R14del*_ can still be partly phosphorylated *in vivo*), mistargeting of mutant PLN to the plasma membrane (Haghighi et al., 2012), and destabilization of pentamers (Haghighi et al., 2006). Although all R14del patients are reported to be heterozygous, explicit accounting for both wild-type and mutant PLN molecules requires a model at least three times the complexity of the current model (due to combinatorial expansion of reactions, molecules, and system equations). Since this exceeds the scope of the present study as well as available data on parameters, we opted for an alternative approach and made qualitative predictions of how known molecular effects of the R14del mutation would individually influence the noise filtering based on the analyses of the original model.

Our qualitative predictions suggested that in a heterozygous setting, mistargeting of R14del PLN, destabilization of pentamers, and potential mutant/wild-type hetero-pentamers would impair both low-pass filtering and bistability (Table S1). Mistargeting and destabilization can be expected to exert a negative effect on noise filtering by reducing the concentration of PLN pentamers. Mutant/wild-type hetero-pentamers would effectively feature fewer phosphorylation sites, thereby reducing the sites that can compete with monomers. As the range of bistability is positively influenced by the number of phosphorylation sites (Ortega et al., 2006), hetero-pentamers can be expected to reduce the bistable range. Thus, the heterozygous R14del situation could be more permissive for short random bursts of PKA activity and lead to higher noise amplitudes, providing an attractive explanation for the susceptibility to cardiac arrhythmias. Since R14del PLN molecules are unresponsive to phosphorylation by PKA, we expect reduced amounts of wild-type pentamers to be the biggest issue for noise filtering.

Although preliminary, our analysis suggests a novel therapeutic strategy: increasing the amount of wild-type pentamers could improve noise filtering and prevent cardiac arrhythmias in patients with the R14del mutation. Potential ways of achieving this include increasing the effective concentration of PLN at the SR, small (and yet to be discovered) molecules that stabilize pentamers without interfering with regulatory enzymes, or metabolically changing the lipid composition of the SR (which regulates PLN pentamerization; cf*.* Zhang et al., 2005).

## Discussion

In the present study, we have demonstrated that at least in our experimental conditions, the buffering effect exerted by PLN pentamers is too moderate and slow to be relevant at the timescale of acute β-adrenergic stimulation. We therefore developed a mathematical model of the PLN regulatory network and studied the role of PLN pentamers in the context of β-adrenergic stimulation from a dynamical systems perspective. Having calibrated the model with own experimental data and experimental parameters from the literature, our simulations predicted delayed phosphorylation responses due to PLN pentamer competition and an inhibitor-1 FFL. Further simulations suggested that PLN phosphorylation could be ultrasensitive and bistable due to cooperative dephosphorylation of PLN pentamers.

**Figure 7:**
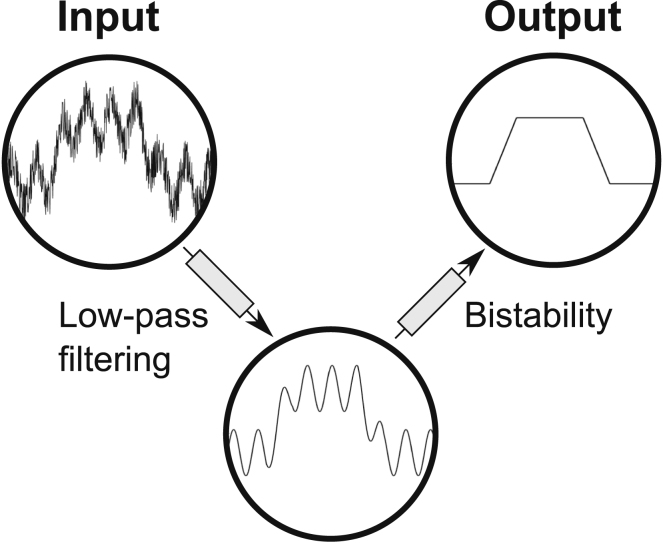
Illustration of proposed noise-filtering principles in the PLN signaling network Low-pass filtering and bistability reject fluctuations at different timescales in a complementary fashion to ensure defined PLN monomer phosphorylation.

Using several different numerical approaches, we have shown that these phenomena can filter out the effect of random fluctuations in PKA activity on PLN monomer phosphorylation; while response delay and persistence sensing constitute a low-pass filter removing fast fluctuations and short stimulus spikes, bistability prevents uncontrolled high-amplitude fluctuations in PLN phosphorylation at critical PKA activity, thereby promoting a well-defined PLN phosphorylation status. Importantly, these noise filters are complementary (Figure 7) and depend largely on PLN pentamers. To our knowledge, this is the first time that a clearly defined physiological advantage of PLN pentamers has been demonstrated.

While we have provided an optimal design for experimentally testing the FFL functionality (e.g., with cell biological approaches), we have confirmed the delay due to pentamer competition *in vitro*, providing experimental evidence for one of the main mechanisms underlying the predicted low-pass filtering. Similar monomer phosphorylation delays due to pentamers have been observed in transfected HEK293 cells, suggesting that the mechanism can operate in living cells (Wittmann et al., 2015). We further replicated ultrasensitive PLN phosphorylation in transfected HEK293 cells, in agreement with previous dose-response data. While the experimental Hill exponents appear lower than predicted, they are still fully consistent with bistability and low-pass filtering (Figure S6). Importantly, we could experimentally confirm that PLN phosphorylation exhibits hysteresis in primary cardiomyocytes as predicted by our model, demonstrating that PLN phosphorylation is bistable.

In an independent study, we show further that the ability of pentamers to shape the response curve of PLN phosphorylation to β-adrenergic stimulation translates into increased dynamic range and sensitivity of cardiac relaxation and is necessary to cope with increased cardiac pressure (data not shown).

Our results also provide a potential explanation for the frequent emergence of cardiac arrhythmias in patients with the R14del mutation. Although preliminary, a first analysis of the molecular consequences of this mutation points toward impaired noise filtering due to reduced amounts of wild-type pentamers. Since the PLN_*R*14*del*_ cardiomyopathy does not respond to conventional heart failure therapy (Eijgenraam et al., 2020), we propose to explore ways to increase the amount of wild-type pentamers in preclinical R14del models as a novel therapeutic approach. A first way to test this concept could be to harness increased pentamerization of the artificial I45A mutant and to determine whether heterozygous R14del/I45A mouse or induced pluripotent stem cell (iPSC) cardiomyocyte models show a lower arrhythmogenic tendency than a heterozygous R14del/wild-type model.

A strength of our model is that it offers new perspectives on multiple and hitherto puzzling phenomena by associating them with a common physiological function: noise filtering. Apart from PLN pentamers, neither their cooperative dephosphorylation (Li et al., 1990; Colyer, 1993) nor the dynamic equilibrium (increased pentamerization upon phosphorylation; Cornea et al., 1997; Hou et al., 2008) were previously known to have clearly defined physiological functions. While pentamers and their cooperative dephosphorylation are necessary in our model for bistability to occur, the dynamic equilibrium makes bistability more robust, potentially by inducing “hidden” feedback loops (Varusai et al., 2015), supporting the emergence of bistability (Figure S7). Interestingly, the recruitment of 14-3-3 proteins to phosphorylated PLN pentamers has recently been found to establish a similar memory of PLN phosphorylation *in vivo* (by slowing down dephosphorylation) and is impaired by R14del (Menzel et al., 2020). This constitutes a double-negative feedback, which may further improve bistability or noise filtering.

Our model also opens a new perspective on the role of inhibitor-1, which is often described as an amplifier for PKA phosphorylation (El-Armouche et al., 2003; Wittköpper et al., 2011). Although this is technically not false, the same level of PLN phosphorylation response could in principle be achieved by simpler means such as reduced SR targeting of PP1 (Alsina et al., 2019). Thus, the delayed response dynamics of the inhibitor-1 FFL and the noise filtering capacity demonstrated in our simulations may be equally important as the influence on steady-state phosphorylation levels.

Noise filtering in the β-adrenergic signaling pathway may only be relevant if the network experiences significant fluctuations of pro-arrhythmogenic potential under some circumstances. While it is known that there is significant electrophysiological variability among individual cardiomyocytes, which can be pro-arrhythmogenic under conditions of reduced cell-cell coupling (Watanabe et al., 1983; Pueyo et al., 2011), a systematic experimental characterization of the noise at multiple nodes of the β-adrenergic signaling network is, to our knowledge, still lacking. However, studies from the 1980s indicate significant fluctuations at baseline, at least in catecholamines (Linsell et al., 1985; Cameron et al., 1987) and aberrant calcium handling or β-adrenergic signaling are well known for triggering cardiac arrhythmias (Landstrom et al., 2017). In general, many perturbations can have both pro- and anti-arrhythmic components, making the emergence of cardiac arrhythmias a complex (Kistamás et al., 2020). While direct stimulation of, for instance, SERCA activity, has been shown to increase the frequency of spontaneous calcium waves (pro-arrhythmogenic), it also increased the threshold for wave occurrence and limited wave propagation (anti-arrhythmogenic), thereby making low SERCA activity potentially pro-arrhythmogenic in the context of β-adrenergic stimulation (Fernandez-Tenorio and Niggli, 2018). Moreover, several studies have indicated PLN to influence repolarization (abnormal T waves in R14del patients (Hof et al., 2019); variants at the PLN gene locus affect QT interval duration (Pfeufer et al., 2009)). It is therefore plausible to assume that large fluctuations in PLN phosphorylation could translate into heterogeneous SERCA activity, potentially generating variability in repolarization behavior, calcium transients, and SR calcium load across cell populations, which may trigger pro-arrhythmic activity such as early or delayed after-depolarizations (Kistamás et al., 2020). In this context, a biochemical memory for PLN phosphorylation such as bistability may prevent individual cardiomyocytes from prematurely returning to a potentially pro-arrhythmogenic low SERCA activity state. As variability on multiple scales ranging from single cells up to whole organisms is increasingly recognized as an important factor for understanding cardiac electrophysiology and arrhythmias (Ni et al., 2018; Gong et al., 2020), noise filtering in the β-adrenergic signaling pathway as an anti-arrhythmic mechanism is a hypothesis worth studying further.

### Further experimental and clinical evidence

In agreement with our results, many proteins of the PLN regulatory network are in fact associated with cardiac arrhythmias by both experimental and clinical data. The natural mutation R9H (Medeiros et al., 2011) has recently been shown to cause ventricular arrhythmias in dogs (Yost et al., 2019), indicating that PLN mutations other than R14del can be arrhythmogenic. Additional evidence that PLN pentamers contribute to noise filtering comes from a mouse model of the natural obscurin variant R4344Q (Hu et al., 2017). Mice carrying this variant developed spontaneous ventricular arrhythmias, which authors attributed to increased SERCA levels and ≈15% fewer pentamers. Although the pathogenicity of this variant is likely restricted to mice (Fukuzawa et al., 2021), the findings support the idea that pentamers can attenuate cardiac arrhythmias.

A similar mechanism may contribute to the pro-arrhythmic effect of thyroid hormones, which increase SERCA and decrease PLN expression (thus decreasing pentamerization) (Tribulova et al., 2020). Apart from PLN, both PP1 and inhibitor-1 have been shown to be involved in the emergence of arrhythmias (Chiang et al., 2016). Reducing the concentration of PP1 at the SR by ablating its targeting subunit PPP1R3A has been shown to lead to atrial fibrillation (Alsina et al., 2019), which is consistent with a smaller bistable region expected from reducing [PP1] in our model. Interestingly, a mouse model of the human inhibitor-1 variant G109E (showing reduced binding to PP1) and mice expressing a constitutively active version of inhibitor-1 developed severe cardiac arrhythmias in response to β-adrenergic stimulation (Haghighi et al., 2015; Wittköpper et al., 2010). Both mutations interfere with the inhibitor-1 FFL and could make PLN phosphorylation more susceptible to noise, according to our model. Contrary to these findings, complete inhibitor-1 ablation has been shown to protect against catecholamine-induced arrhythmias (El-Armouche et al., 2008), which led to a debate over whether inhibitor-1 is pro- or anti-arrhythmogenic (Nicolaou et al., 2009; Wittköpper et al., 2011). Settling this debate may require a nuanced answer distinguishing between pro- and anti-arrhythmogenic effects (e.g., pro-arrhythmogenic PKC phosphorylation sites versus noise filtering). Moreover, the complete loss of inhibitor-1 may be compensated for by upregulating FFLs involving, for example, Hsp20 (Kranias and Hajjar 2012).

An intriguing line of evidence that noise filtering may also be upregulated in response to arrhythmic heart activity came from a recent study on arrhythmogenic cardiomyopathy (ACM) patients. In ACM patients without PLN mutation, PLN protein expression was shown to be upregulated more than 2-fold, which the authors hypothesized to be a yet-to-be-elucidated compensatory mechanism (Akdis et al., 2016). As higher PLN concentration leads to increased pentamerization due to mass action, both noise-filtering functions predicted by our model would be enhanced, providing an attractive explanation for this observation.

These studies show that perturbing components that contribute to noise filtering in our model can lead to cardiac arrhythmias, whereas enhancing their functionality may protect against other pro-arrhythmogenic factors.

### Limitations and conclusions

Like every modeling study, we had to rely on simplifying assumptions at several points during model development. For example, our model only accounts for a subset of interactions with PLN that we deemed most relevant for our purpose; it assumes that the modeled processes are described well enough by ordinary differential equations, even though much of the biochemistry takes place on the two-dimensional SR surface; parameters and species concentrations of our model come from different sources (e.g., fitted to our own experimental data, directly measured or fitted parameters from the literature). Furthermore, we have considered noise only in terms of fluctuating PKA activity (representing the input of our model), although extrinsic and intrinsic noise sources affect all molecular processes whose full exploration requires stochastic simulations (Johnston, 2012; Tsimring, 2014). Due to the medical relevance of the R14del mutation, perhaps the most important limitation to highlight is that our analysis of the R14del mutation is preliminary. Since this mutation has been shown to alter additional interactions not currently represented in our model, the model does not yet capture other pathophysiological processes such as (potentially pro-arrhythmogenic) cardiac remodeling (Te Rijdt et al., 2016).

While we have partly addressed some limitations, for example, by performing complementary analyses or systematic explorations of the parameter space, others will need to be addressed in future experimental and theoretical investigations. Despite these limitations, the experimental data support the conclusions from our simulations. We believe our model thus offers a novel and exciting perspective on the physiological role of PLN pentamers and will prove to be a useful starting point for further investigations.

## STAR★Methods

### Key resources table

**Table:** 

REAGENT or RESOURCE	SOURCE	IDENTIFIER
**Antibodies**		

Phospholamban (PLN, PLB) (pSer16) pAb	Badrilla	Product code: A010-12AP, RRID: AB_2617047
Phospholamban (PLN, PLB) mAb (clone A1)	Badrilla	Product code: A010-14, RRID: AB_2617049
Goat polyclonal anti-rabbit, HRP coupled	Jackson	Cat# 111-035-003, RRID: AB_2313567
Goat-anti-rabbit IgG (H+L), HRP coupled	Thermo Fischer	Cat# 31460, RRID: AB_228341
Goat-anti-mouse IgG (H+L), HRP coupled	Thermo Fischer	Cat# 31430, RRID: AB_228307

**Chemicals, peptides, and recombinant proteins**		

Clarity™ ECL Western Substrate	Bio-Rad	Cat# 1705060
Quick Coomassie Stain	Generon	Cat# NB-45-00078-1L
Pro-Q™ Diamond Stain	Thermo Fischer	Cat# P33300
Oriole™ Fluorescent Gel Stain	Bio-Rad	Cat# 161-0496
Ponceau S solution	Sigma-Aldrich	Cat# P7170
Geltrex LDEV-Free, hESC-Qualified, Reduced Growth Factor Basement Membrane Matrix	GIBCO	Cat# A1413302
Human PLN and AFA-PLN peptides	Pepscan	N/A
Collagenase, Type 2	Worthington	Cat# LS004176
Pancreatin	Sigma-Aldrich	Cat# P3292-25G
Dulbecco’s Modified Eagle’s Medium (DMEM)	Sigma-Aldrich	Cat# D5030
M199	Sigma-Aldrich	Cat# M4530
Fetal Calf Serum (FCS), heat inactivated	Sigma-Aldrich	Cat# F4135
Horse Serum (HS)	Sigma-Aldrich	Cat# H1270
Isoproterenol-hydrochlorid	Sigma-Aldrich	Cat# I5627-25G
Forskolin	Santa Cruz	Cat# sc-3562
Staurosporine	Cambridge Bioscience	Cat# SM97-5
PhosSTOP Inhibitor Cocktail Tablets	Roche	Cat# 04906837001
cOmplete, EDTA-free Protease Inhibitor Cocktail Tablets	Roche	Cat# 11873580001
Benzonase® Nuclease, Purity > 90%	Milipore	Cat# 70746-4
PKA, Catalytic Subunit, Bovine Heart	Sigma-Aldrich (Gift from Dr Thomas Kampourakis)	Cat# 539576

**Experimental models: cell lines**		

HEK293 cells	ATCC	CRL1573

**Experimental models: organisms/strains**		

*Rattus norvegicus* Wistar outbred pups. Strain: Hsd:WI	Envigo	Order code: 001; RRID: RGD_737960

**Recombinant DNA**		

pCDNA-PLN	Wittmann et al. 2015	N/A

**Software and algorithms**		

MATLAB R2019b	MathWorks	https://www.mathworks.com/
COPASI v4.26	(Hoops et al., 2006)	http://copasi.org/
GraphPad Prism v8.3	GraphPad Software, Inc.	https://www.graphpad.com
ImageLab v6.0	Bio-Rad	https://www.bio-rad.com/en-us/product/image-lab-software
Bifurcation diagrams	Daniel Koch	https://www.ebi.ac.uk/biomodels/MODEL1910220002
Model code	Daniel Koch	https://www.ebi.ac.uk/biomodels/MODEL2011110001

**Other**		

4–20% Mini-PROTEAN® TGX™ Precast Protein Gels	Bio-Rad	Cat# 4561096
μ-Dish 35 mm, cell culture dish	Ibidi	Cat# 81156

### Resource availability

#### Lead contact

Further information and requests for resources should be directed to and will be fulfilled by the lead contact, Daniel Koch (dkoch.research@protonmail.com).

#### Materials availability

This study did not generate new unique reagents.

### Experimental model and subject details

#### Cell culture

Neonatal ventricular rat cardiomyocytes (NRCs) were isolated from Wistar rat pups and cultured as described previously (Simpson and Savion, 1982; Zhang et al., 2006). In brief, hearts were isolated from Wistar rat pups at postnatal day 0 to 2 and cut into 4 in ice cold ADS (116 mM NaCl, 20 mM HEPES, 0.8 mM NaH_2_PO_4_, 5.6 mM glucose, 5.4 mM KCl, 0.8mM MgSO_4_). The hearts were enzymatically digested in a sequential manner by incubation in enzyme solution containing collagenase type II (57.5 U/ml) and pancreatin (1.5 mg/ml) for 4-5 times for 15 min in a shaking incubator at 37^°^C. The supernatant is collected into medium containing 5% FCS and passed through a 70 micron cell strainer (Falcon Corning) before being pelleted at low speed. The cells were pre-plated onto Nunc dishes in plating medium (DMEM, 5% FCS, 10% HS, non-essential amino acids, penicillin/streptomycin (P/S) and L-glutamine) for 2 h to allow non-myocytes to adhere. The non-adherent cardiomyocyte enriched fraction is then plated onto Geltrex (GIBCO) coated ibi-treat 35 mm dishes (Ibidi) and cultured at 37^°^C and 5% CO_2_. Once the cells recovered (2-3 days), the non-adherent cells were washed away with culture medium (M199, DBSSK [116mM NaCl, 1 mM NaH_2_PO_4_, 0.8 mM MgSO_4_, 32.1 mM NaHCO_3_, 5.5 mM glucose, 1.8 mM CaCl_2_ pH7.2], 4% Horse serum, P/S and L-glutamine) and cultured until day 8-9 for further maturation (medium exchange every 2-3 days).

HEK293 cells were cultured in DMEM medium supplemented with 10%FCS and P/S at 37^°^C and 5% CO_2_ and not used beyond passage 15.

### Method details

#### Experimental procedures

##### Reconstitution of PLN in detergent micelles

PLN (human wild-type and monomeric ‘AFA’ mutant C36A, C41F, C46A) was purchased as a synthetic peptide from Pepscan (Lelystad, Netherlands). Successful synthesis of the peptide, as indicated by the correct molecular weight, was confirmed by Pepscan via mass-spectrometry (Figure S8A). For further analysis, 1 mg of lyophilized peptide was resolubilized in 1 ml of buffer for 2 h at room temperature under gentle overhead agitation (10 rpm) and subsequently centrifuged in a bench-top centrifuge for 10 min at >10000 ×g, 4^°^C to get rid of insoluble residual material. For experiments involving PLN phosphorylation, we tested various detergents in a buffer with physiological ionic strength and pH and found that Triton X-100, which is a milder detergent than the harsh ionic SDS, is excellent at solubilizing PLN at rather low detergent concentrations and allows for effective pentamerization as well as rapid phosphorylation by PKA. We thus used the following Triton X-100 based buffer (TBB) for all experiments involving PLN phosphorylation: 50 mM TRIS-HCl pH 7.5, 100 mM NaCl, 10% (v/v) glycerol, 0.5% (v/v) Triton X-100, 2 mM DTT.

Interestingly, although showing less pentamerization than wild-type PLN, AFA-PLN was not entirely monomeric when kept on ice or even at room temperature. However, short heating for 15 min at 50^°^C was sufficient to dissociate all pentamers into monomers (Figure S8B). Although some smearing of band was visible after SDS-PAGE, no precipitation in solution was visible even after 45 min at 50^°^C and AFA-PLN still appears to be an excellent substrate for PKA after heating, which indicates that heating PLN for 15 min at 50^°^C does not seriously denature the protein. Once dissociated, no significant re-oligomerization was observed after incubation at 25^°^C for 2 h (the time frame for our phosphorylation experiments) or after snap-freezing and quick thawing.

##### Semi-native SDS-PAGE

PLN forms stable pentamers even in harsh SDS sample buffer which only dissociate upon sample boiling. As SDS sample buffer is likely to interfere with less stable proteins (e.g., enzymes), all phosphorylation reactions were performed in TBB and analyzed using semi-native SDS-PAGE by directly applying the native samples (e.g., phosphorylated or unphosphorylated PLN in TBB) into the wells of the gels (4%–20% Mini-PROTEAN® TGX™ Precast Protein Gels, Bio-Rad) without addition of other buffers or boiling. All samples contained 10% glycerol to allow samples to settle into the wells and to avoid mixing with running buffer.

##### Oriole staining and quantification of absolute concentrations

Since conventional Coomassie staining of protein gels only has limited sensitivity and more sensitive silver-staining protocols are typically not suitable for quantitative purposes, we used Oriole™ Fluorescent Gel Stain for detection and quantification of PLN (Bio-Rad). Staining was performed for 90 min according to the manufacturer’s instructions. Gels were imaged on a ChemiDocTM XRS+ imaging system (Bio-Rad). The method shows a wide linear range for PLN and is neither affected by oligomerization or phosphorylation status of PLN (Figures S8C–S8E). Since the Oriole™ signal is not affected by oligomeric state, the signal for an oligomeric species is directly proportional to the number protomers in a complex. Knowing the total PLN concentration of a sample thus allows to calculate the absolute concentrations of monomers and pentamers by [PLN_*x*_] = $\frac{F_{PLN_{x}}^{Oriole}\cdot \left[PLN_{tot}\right]}{x}$, where $F_{PLN_{x}}^{Oriole}$ is the fraction which PLN_*x*_ contributes to the total Oriole™ signal. In either SDS-sample buffer or TBB we observed no other PLN forms than monomers and pentamers and occasionally a weak band for dimers at higher concentrations. Since the dimer band was not well demarked and negligible compared to the monomer and pentamer bands, we did not quantify dimers.

##### Dilution experiments

Samples were diluted in TBB or SDS with end concentrations ranging from ≈ 0.96 mg/ml (undiluted) to 5 μg/ml in a final volume of 40 μl. After dilution, samples were left to pre-equilibrate for 45 min at room temperature before being incubated in a PCR machine for 75 min at 37^°^C with heated lid at 50^°^C to avoid evaporation. To avoid disturbing the pentamerization equilibrium by sample cooling, semi-native SDS-PAGE was performed using SDS-running buffer pre-warmed to 37^°^C. Total protein amount for each well was within the determined linear range.

For production of phosphorylated PLN, TBB samples were supplemented with 250 μM ATP, 5 mM MgCl_2_ and 3 U/μl PKA (Sigma-Aldrich). Phosphorylation was allowed to proceed for 16 h at 4^°^C and given another hour at room temperature to approach completion. To avoid differences due to ionic strength, precipitation or evaporation, samples for unphosphorylated PLN in TBB were treated accordingly, but without addition of PKA.

##### Dissociation time course experiments

To determine pentamer dissociation dynamics, 40 μl of 0.96 mg/ml PLN in TBB supplemented with 250 μM ATP and 5 mM MgCl_2_ were pre-equilibrated for 30 min at RT followed by 30 min at 37^°^C. Samples were diluted 20-fold with 37^°^C pre-warmed TBB and incubated for up to 15 min at 37^°^C. To simultaneously determine the oligomeric status at different time points, dilutions were started in replicates at 1.5, 7.5 and 15 min before semi-native SDS-page (performed with 37^°^C pre-warmed running buffer). Samples were processed and loaded to the gel using a multichannel pipette to minimize sample processing time.

##### *In vitro* phosphorylation time courses of PLN by PKA

To determine the competitive effect of pentamers on monomer phosphorylation, we compared the phosphorylation dynamics of wild-type PLN and AFA-PLN. To ensure complete dissociation into monomers, AFA-PLN was heated to 50 ^°^C for 15 min followed by 10 min incubation at 25 ^°^C immediately before the experiment. Phosphorylation reactions were performed at 25^°^C in a volume of 150 μl TBB supplemented with 250 μM ATP, 5 mM MgCl_2_ and 6.25 U/μl (≈ 7.7 nM) PKA (Sigma-Aldrich) with an end concentration of 0.962 mg/ml (≈ 157 μM) wild-type PLN or 0.32 mg/ml AFA-PLN (at which there is an equimolar monomer concentration of ≈ 52 μM between wild-type PLN and AFA-PLN). Samples (8 μl) were taken at 0, 5, 15, 30, 60, 90 and 120 min, snap-frozen and stored in liquid nitrogen until separation by semi-native SDS-PAGE (1.5 μl/well for wild-type PLN, 2 μl/well for AFA-PLN). Phosphorylation was detected using Pro-Q™ Diamond staining (Thermo Fisher) according to the manufacturer’s instructions (1 h staining step), followed by Quick Coomassie Staining (Generon) to visualize total protein amount. Gels were imaged on a ChemiDocTM XRS+ imaging system (Bio-Rad) and data were quantified using the ImageLab v6.0 software (Bio-Rad). All monomer phosphorylation data were in the linear range of the Pro-Q™ Diamond stain. The phosphorylation signal of each experiment was corrected by total protein amount as given by the Coomassie signal and by subtracting the background signal at 0 min. Relative phosphorylation levels were calculated by fitting progress curves of wild-type pentamers and AFA-PLN_1_ to a hyperbola ($Y\left(t\right)=S_{max}\text{∗}t/\left(K+t\right)$ and dividing the corrected phosphorylation signal by $S_{max}$ (the signal expected for complete phosphorylation). Since phosphorylation of wild-type monomers was still linear during the probed reaction period (preventing reliable fitting to a hyperbola), relative phosphorylation was calculated by using $S_{max}$ from wild-type pentamers scaled by r = 52 μM / (157 - 52) μM, the amount of phosphorylation sites in monomers relative to the amount of phosphorylation sites in pentamers at the given total concentration of PLN.

##### PKA-dependent phosphorylation of PLN in transfected HEK293 cells (Schmitt lab)

pcDNA3-PLN was expressed in HEK293 cells as described previously (Wittmann et al., 2015). PLN-expressing cells were then treated for 40 min with forskolin (Santa Cruz) in DMEM medium at 37^°^C at different concentrations to induce PKA-dependent phosphorylation of PLN. Cells were washed with PBS before mechanical lysis in PBS containing protease inhibitors and phosphatase inhibitors. Lysates were centrifuged for 20 min at > 10,000 × g at 4^°^C and supernatants were used for western blot analysis. Equal amounts of protein (Pierce® BCA Assay Kit, ThermoScientific) were separated on 15% polyacrylamide gels and transferred to PVDF membranes (Immobilon®-P, Millipore) before overnight incubation with primary antibodies in TBS-T / 5% milk (10 mM Tris, 150 mM NaCl, 0.1% Tween 20, pH 7.6, 5% milk) at 4^°^C. Antigen detection was performed by chemiluminescence using secondary antibodies coupled to horseradish peroxidase (Thermo Fischer) and Luminata Forte Western HRP substrate (Millipore). The following antibodies were used for detection of proteins: anti-PLN (A1, Badrilla, 1:5000 dilution), anti-phospho-PLN (Ser16, Badrilla, 1:5000 dilution). Data were quantified using the ImageLab v6.0 software (Bio-Rad).

##### Hysteresis detection of PKA-dependent PLN phosphorylation in neonatal rat cardiomyocytes

Hysteresis is defined as the dependence of the state of a system on the history of that system. A common approach for detecting hysteresis in a process (‘output’) which is triggered by a certain stimulus (‘input’) is therefore to compare the steady state output at a certain input level when the system had no previous input versus when the system relaxes from a much higher input level: if the output is different, the system exhibits hysteresis. When designing experiments aimed at detecting hysteresis, however, it is important to consider the timescales at which the process under investigation reaches steady state in terms of the time to respond to stimulation, and in terms of the relaxation time after stimulus removal.

PLN phosphorylation in response to β-adrenergic stimulation of intact rat hearts with isoprotenorol has been demonstrated to rapidly reach steady state within 1 min both at half-maximal and maximal stimulation (Kuschel et al., 1999). Similarly, dephosphorylation of PLN in isoprotenerol stimulated rat hearts after stimulus removal has been reported to be complete within 3 min (Talosi et al., 1993). In agreement with these studies, we found PLN phosphorylation upon 1 μM isoprotenorol stimulation in isolated NRCs to be complete within 1 min and dephosphorylation after isoprotenorol removal to be complete within 2 min (data not shown).

To test for hysteresis in PLN phosphorylation, 8-9 day old NRCs were treated according to either a ‘going up’ or ‘coming down’ protocol. *Going up:* all medium was removed from the dishes and replaced with 1 mL culture medium containing the experimental target concentration of isoprotenorol ([ISO]). After incubation for 5 min at 37^°^C, cells were washed in 1 mL PBS + target [ISO] (at room temperature) before all liquid was thoroughly removed. 18 μl of ice cold lysis buffer were added to the cells (50 mM Tris pH 7.5, 100 mM NaCl, 1mM MgCl_2_, 2 mM DTT, 0.5% (v/v) Triton x-100, 10% Glycerol, 2x PhosSTOP, 0.5 μM Staurosporine, 1x cOmplete, EDTA-free Protease Inhibitor Cocktail, Benzonase 1.5 μl / ml buffer). Lyzed cells were directly scraped off the dish with a bent 200 μl pipette tip and snapfrozen in liquid nitrogen or briefly stored on ice until the dish was processed. *Coming down:* all medium was removed from the dishes and replaced with 1 ml culture medium containing 1 μM [ISO] in which cells were incubated for 2 min at 37^°^C for pre-stimulation. After pre-stimulation, all medium was removed and cells were washed for 1 min in 1.5 ml culture medium at target [ISO]. After washing out the excess ISO, the medium was replaced again with 1 ml new culture medium at target [ISO] and cells were incubated for 5 min at 37^°^C to reach steady state before removing the medium and washing the cells in 1 mL PBS + target [ISO] at room temperature. 18 μl of ice cold lysis buffer were added to the cells. Lyzed cells were directly scraped off the dish with a bent 200 μl pipette tip and snapfrozen in liquid nitrogen or briefly stored on ice until the dish was processed.

Note: the washing step after pre-stimulation is essential and particular care needs to be taken to remove *all* liquid from the dishes after pre-stimulation and washing in order to ensure that non-pre-stimulated and pre-stimulated cells are exposed to the same target [ISO] at steady state. To minimize the impact of environmental parameters such as temperature, dishes were handled on a heating pad at 37^°^C and medium containing target [ISO] was pre-warmed and kept in 37^°^C warm water until use. To minimize experimental variation, the same batch of target [ISO] adjusted culture medium was used for non-pre-stimulated and pre-stimulated cells.

Cell lysates were pelleted by centrifugation for 5 min at >10000 ×g, 4^°^C before 13 μl of the soluble fraction were separated by semi-native SDS-PAGE followed by wet transfer of the proteins to a nitrocellulose membrane (GE Healthcare) at 100V constant current for 45 min in blotting buffer (3 g/l Tris, 14.5 g/l glycine, 0.1 g/l SDS, 20% (v/v) ethanol). After blotting, membranes were stained with Ponceau S solution to normalize for total protein amount and to confirm successful transfer. Unspecific binding sites on the membrane were blocked for 30 min at room temperature in low-salt binding buffer / 5% milk (10 g/l Tris pH 7.4, 9 g/l NaCl, 1% (v/v) Tween-20, 5% (w/v) milk powder) before the membrane was incubated overnight with primary antibodies (anti-pSer16 PLN, 1:4000 dilution) at 4^°^C.

Antigen detection was performed by chemiluminescence using secondary antibodies coupled to horseradish peroxidase (goat anti-rabbit pAb, Jackson, 1:1000 dilution) and Clarity ECL Western Substrate (Bio-Rad) on a ChemiDocTM XRS+ imaging system (Bio-Rad). Data were quantified using the ImageLab v6.0 software (Bio-Rad). All data were in the combined linear range. For analysis, pSer16-PLN signals were corrected by the total protein signal and normalized to the maximum value. To visualize data from different membranes on the same plot, data was scaled by setting the total ‘going up’-signal at 5 nM target [ISO] to 0.5 and at 1 μM target [ISO] to 1. Statistical comparisons were only made between signals from the same membrane.

#### Development of the mathematical model

In order to develop a mathematical model of the PLN regulatory network, we first needed to find a mathematical description of how PLN monomers assemble into pentamers. Therefore, we considered several mass action kinetics based possibilities.

##### A mass action kinetics model of PLN pentamer assembly

The assembly of PLN monomers into pentamers likely follows either of three possible pathways depicted in the scheme on the left side of Figure S9. While model 1 considers all reaction routes possible, model 2 and model 3 assume pentamer assembly to follow a monomer→dimer→tetramer →pentamer or monomer→dimer→trimer→ pentamer pathway, respectively. Dimers, trimers and tetramers have been reported *in vitro* (see e.g., Reddy et al., 1995), but monomers and pentamers are typically the predominantly observed molecular species (including the present study). While this implies that PLN oligomers with < 5 protomers are usually low abundant and transient species, it is difficult to decide between any of the three pathways on *a priori* grounds. We thus formulated mass-action kinetics models for each possibility (Figure S9).

While the schemes for model variant 2 and 3 are linear reaction routes, model 1 contains two cycles. For thermodynamic reasons, model 1 must thus obey the following relations between equilibrium constants:

(Equation 1) $$K_{2,3}K_{3,5}=K_{2,4}K_{4,5}$$

(Equation 2) $$K_{1,2}K_{2,4}=K_{2,3}K_{3,4}$$

(Equation 3) $$K_{1,2}K_{3,5}=K_{3,4}K_{4,5}$$

Replacing equilibrium constants with the rate constants shown in Figure S9 yields:

(Equation 4) $$\frac{k_{3}k_{11}}{k_{4}k_{12}}=\frac{k_{7}k_{9}}{k_{8}k_{10}}$$

(Equation 5) $$\frac{k_{1}k_{9}}{k_{2}k_{10}}=\frac{k_{3}k_{5}}{k_{4}k_{6}}$$

(Equation 6) $$\frac{k_{1}k_{11}}{k_{2}k_{12}}=\frac{k_{5}k_{7}}{k_{6}k_{8}}$$

For parameter estimation, $k_{7}$ and $k_{5}$ were assigned by solving Equations 4 and 5, respectively. Equation 6 was used as an additional constraint on the parameter space.

After setting up the constraints for model 1, we used the data from our dilution and dissociation time course experiments for calibration of all three models. We found that all three models can reproduce the dissociation time course sufficiently (Figure S10A). Model 1 and model 2 show generally good agreement with the data from dilution experiments apart from small deviations from the measured pentamer concentrations at the lower range of total concentrations (Figure S10B). Model 3, on the other hand, shows a systematic deviation from most experimentally measured pentamer concentrations. Simulated steady-state concentrations of monomers and pentamers are fairly similar for all three model variants (Figure S10C), indicating that the shortcoming of model 3 is a mismatch at the timescale of hours. Also note that at steady state (which in this case is identical to the equilibrium of the reaction) and at [PLN]_*tot*_ > 100 μM, there is effective monomer buffering (Figure S10C, red curves).

Moreover, model 3 predicts high trimer concentrations at steady state which should have been clearly visible in our experimental conditions. Taken together, this indicates that model 3, solely relying on the monomer→dimer→trimer→pentamer pathway, cannot account for our experimental data. In contrast, although the available data are not sufficient to identify individual rate constants (Figure S10D), we conclude that both model 1 and 2 can faithfully reproduce most of our experimental data. To choose between models (using their best fit parameter sets), we applied the Akaike Information Criterion (AIC) which ranked model 2 as best performing (AIC scores: model 1 = −735.2, model 2 = −741.2, model 3 = −735.9). We, therefore, used model 2 as the basis for the extended model in the remainder of this study.

Before expanding the model, we used our quantitative description of the pentamerization reaction to re-evaluate some of the numbers published on what the effective concentration of PLN in the SR membrane might be and what fraction of SERCA will be occupied at this concentration. In SDS-PAGE analyses of cell/tissue homogenates, 75%–90% of PLN is pentameric (MacLennan and Kranias 2003; Kimura et al., 1997). If oligomerization parameters of PLN in the SR membrane are similar to those in detergent micelles, this would require PLN concentrations of 200 to 600 μM *in vivo* (Figure S11). Although this appears to be very high, PLN is known to be very abundant (*cf.* Table: review protein concentrations) and the required number of molecules to reach high *effective concentrations* is likely lower on a two-dimensional surface such as the SR membrane than in solution (Abel et al., 2012). Moreover, a PLN concentration of ≈ 250 μM would result in a fractional SERCA occupation of about 40%–60% according to published $K_{d}$ values. This fits well to the observation that about 40% of SERCA activity is functionally regulated by PLN (Brittsan et al., 2000). At a total PLN concentration of 250 μM, the concentration of monomers is ≈ 58 μM, the concentration of pentamers ≈ 38 μM, i.e., about 76% of PLN molecules would be in a pentameric complex. *In vivo*, oligomerization of PLN happens in the SR lipid bilayer, an approximately two-dimensional surface. Thus, PLN exists not in a well-mixed solution and its concentration is rather given by molecules per area. For our purposes, however, we assume these processes can be approximated by an effective PLN concentration which allows us to formulate the model with ODEs, similar to previous models (Saucerman et al., 2003).

##### Full phospholamban model in the context of β-adrenergic stimulation

As outlined in the main text, we extended the mass action kinetics model of pentamerization by accounting for PLN phosphorylation at serine 16. Phosphorylation of phospholamban leads to a combinatorial expansion of oligomeric phospho-isoforms for which we denote the number phosphorylated subunits by a superscript (pX), e.g., $PL{N_{1}}^{\left(p0\right)}$ for unphosphorylated monomers or$PL{N_{5}}^{\left(p3\right)}$ for pentamers with 3 phosphorylated subunits etc. We further included reactions and molecules with a well established role in regulating PLN phosphorylation at serine 16, i.e., enzymes PKA, PP1, PP2A and inhibitor-1. Figure S12A shows the complete reaction scheme of our model. Before substituting rate identifiers with their rate laws, the model equations are as follows:

##### Model equations

$$\frac{d}{dt}\left[PLN_{1}^{\left(p0\right)}\right]\left(t\right)=2v_{2}+v_{4}+v_{20}+v_{24}+v_{28}+v_{32}+v_{36}+v_{40}+v_{41}$$

$$-2v_{1}-v_{3}-v_{19}-v_{23}-v_{27}-v_{31}-v_{35}-v_{39}$$

$$\frac{d}{dt}\left[PLN_{2}^{\left(p0\right)}\right]\left(t\right)=v_{1}+2v_{8}+v_{10}+v_{12}-v_{2}-2v_{7}-v_{9}-v_{11}$$

$$\frac{d}{dt}\left[PLN_{2}^{\left(p1\right)}\right]\left(t\right)=v_{3}+v_{10}+2v_{14}+v_{16}-v_{4}-v_{9}-2v_{13}-v_{15}$$

$$\frac{d}{dt}\left[PLN_{2}^{\left(p2\right)}\right]\left(t\right)=v_{5}+v_{12}+v_{16}+2v_{18}-v_{6}-v_{11}-v_{15}-2v_{17}$$

$$\frac{d}{dt}\left[PLN_{4}^{\left(p0\right)}\right]\left(t\right)=v_{7}+v_{20}+v_{22}-v_{8}-v_{19}-v_{21}$$

$$\frac{d}{dt}\left[PLN_{4}^{\left(p1\right)}\right]\left(t\right)=v_{9}+v_{24}+v_{26}-v_{10}-v_{23}-v_{25}$$

$$\frac{d}{dt}\left[PLN_{4}^{\left(p2\right)}\right]\left(t\right)=v_{11}+v_{13}+v_{28}+v_{30}-v_{12}-v_{14}-v_{27}-v_{29}$$

$$\frac{d}{dt}\left[PLN_{4}^{\left(p3\right)}\right]\left(t\right)=v_{15}+v_{32}+v_{34}-v_{16}-v_{31}-v_{33}$$

$$\frac{d}{dt}\left[PLN_{4}^{\left(p4\right)}\right]\left(t\right)=v_{17}+v_{36}+v_{38}-v_{18}-v_{35}-v_{37}$$

$$\frac{d}{dt}\left[PLN_{5}^{\left(p0\right)}\right]\left(t\right)=v_{19}+v_{43}+v_{44}-v_{20}-v_{42}$$

$$\frac{d}{dt}\left[PLN_{5}^{\left(p1\right)}\right]\left(t\right)=v_{21}+v_{23}+v_{42}+v_{46}+v_{47}-v_{22}-v_{24}-v_{43}-v_{44}-v_{45}$$

$$\frac{d}{dt}\left[PLN_{5}^{\left(p2\right)}\right]\left(t\right)=v_{25}+v_{27}+v_{45}+v_{49}+v_{50}-v_{26}-v_{28}-v_{46}-v_{47}-v_{48}$$

$$\frac{d}{dt}\left[PLN_{5}^{\left(p3\right)}\right]\left(t\right)=v_{29}+v_{31}+v_{48}+v_{52}+v_{53}-v_{30}-v_{32}-v_{49}-v_{50}-v_{51}$$

$$\frac{d}{dt}\left[PLN_{5}^{\left(p4\right)}\right]\left(t\right)=v_{33}+v_{35}+v_{51}+v_{55}+v_{56}-v_{34}-v_{36}-v_{52}-v_{53}-v_{54}$$

$$\frac{d}{dt}\left[PLN_{5}^{\left(p5\right)}\right]\left(t\right)=v_{37}+v_{54}-v_{38}-v_{55}-v_{56}$$

$$\frac{d}{dt}\left[Inh-1^{\left(p0\right)}\right]\left(t\right)=v_{58}-v_{57}$$

$$\frac{d}{dt}\left[PP1\right]\left(t\right)=v_{60}-v_{59}$$

We do not account for synthesis and degradation of proteins and thus assume the total amounts of PLN, inhibitor-1 and PP1 to be conserved. This allows us to determine three species by the following algebraic equations:

$$\left[PLN_{1}^{\left(p1\right)}\right]\left(t\right)={\left[PLN\right]}_{tot}-\left[PLN_{1}^{\left(p0\right)}\right]\left(t\right)-2\left(\left[PLN_{2}^{\left(p0\right)}\right]\left(t\right)+\left[PLN_{2}^{\left(p1\right)}\right]\left(t\right)+\left[PLN_{2}^{\left(p2\right)}\right]\left(t\right)\right)$$

$$-4\left(\left[PLN_{4}^{\left(p0\right)}\right]\left(t\right)+\left[PLN_{4}^{\left(p1\right)}\right]\left(t\right)+\left[PLN_{4}^{\left(p2\right)}\right]\left(t\right)+\left[PLN_{4}^{\left(p3\right)}\right]\left(t\right)+\left[PLN_{4}^{\left(p4\right)}\right]\left(t\right)\right)$$

$$-5\left(\left[PLN_{5}^{\left(p0\right)}\right]\left(t\right)+\left[PLN_{5}^{\left(p1\right)}\right]\left(t\right)+\left[PLN_{5}^{\left(p2\right)}\right]\left(t\right)+\left[PLN_{5}^{\left(p3\right)}\right]\left(t\right)+\left[PLN_{5}^{\left(p4\right)}\right]\left(t\right)+\left[PLN_{5}^{\left(p5\right)}\right]\left(t\right)\right)$$

$$\left[PP1:Inh-1^{\left(p1\right)}\right]\left(t\right)={\left[PP1\right]}_{tot}-\left[PP1\right]\left(t\right)$$

$$\left[Inh-1^{\left(p1\right)}\right]\left(t\right)={\left[Inh-1\right]}_{tot}-\left[Inh-1^{\left(p0\right)}\right]\left(t\right)-\left[PP1:Inh-1^{\left(p1\right)}\right]\left(t\right),$$

where ${\left[PLN\right]}_{tot},{\left[PP1\right]}_{tot}$ and ${\left[Inh-1\right]}_{tot}$ are the conserved total concentrations of PLN, PP1 and inhibitor-1, respectively.

##### Other model quantities (relative phosphorylation levels)

For the sake of clarity, only relative phosphorylation levels of PLN monomers and pentamers, defined as $PLN_{1}^{rp}=\frac{\left[PLN_{1}^{\left(p1\right)}\right]}{\left[PLN_{1}^{\left(p0\right)}\right]+\left[PLN_{1}^{\left(p1\right)}\right]}$ and $PLN_{5}^{rp}=\frac{\left[PLN_{5}^{\left(p1\right)}\right]+2\left[PLN_{5}^{\left(p2\right)}\right]+3\left[PLN_{5}^{\left(p3\right)}\right]+4\left[PLN_{5}^{\left(p4\right)}\right]+5\left[PLN_{5}^{\left(p5\right)}\right]}{5\left(\left[PLN_{5}^{\left(p0\right)}\right]+\left[PLN_{5}^{\left(p1\right)}\right]+\left[PLN_{5}^{\left(p2\right)}\right]+\left[PLN_{5}^{\left(p3\right)}\right]+\left[PLN_{5}^{\left(p4\right)}\right]+\left[PLN_{5}^{\left(p5\right)}\right]\right)}$, respectively, have been plotted.

#### Reaction rates

##### Oligomerization of PLN

Combinatorial expansion due to phospho-isoforms of oligomeric complexes can lead to thermodynamic inconsistencies if mass action kinetics are applied to the oligomerization reactions as given in a reaction scheme. This can be circumvented by introducing balancing coefficients which account for the effective oligomerization rates regardless of combinatorial effects. We thus modeled oligomerization reactions with balanced mass action kinetics as described in Koch (2020).

##### Dynamic equilibrium of PLN

How exactly dynamic equilibrium of PLN works on the molecular level is currently not known, although electrostatic interactions (Cornea et al., 1997; Hou et al., 2008) and anionic detergents seem to play an important role. Since electrostatic interactions can increase the association rate constant of an interaction (Schreiber et al., 2009), we assumed this to be the case for phosphorylated PLN. We, therefore, introduced a dimensionless control parameter ω, which increases association rate constants of oligomerization reactions in an exponential fashion depending on how many phosphorylated subunits are involved.

##### Enzymatic reactions catalyzed by PKA, PP1 and PP2A

Reactions catalyzed by PKA, PP2A were modeled using a modified Michaelis-Menten rate law, which accounts for competition between multiple substrates $S_{1},\ldots ,S_{n}$:

$$v_{i}=\frac{V_{max}S_{i}}{K_{m_{i}}\left(1+\sum_{j\in J\setminus \left\{i\right\}}\frac{S_{j}}{K_{m_{j}}}\right)+S_{i}},$$

where J={1,…,n}, and $v_{i}$ describes the rate of consumption of substrate $S_{i}$ (Schäuble et al., 2013). Since PLN pentamers and monomers are several orders of magnitude more abundant than dimers or tetramers, the possibility of direct dimer or tetramer (de-)phosphorylation can be ignored and was thus not accounted for in this study. Studying the mechanisms of phosphorylation and dephosphorylation of PLN in native SR membrane preparations, (Li et al., 1990) found that the distribution of pentameric phospho-isoforms after stimulation of PKA follows a binomial pattern, suggesting that PKA utilizes a non-cooperative random mechanism for phosphorylation of PLN pentamers (Li et al., 1990). In contrast, completely unphosphorylated PLN pentamers were the first species to accumulate during dephosphorylation, which led to a strongly U-shaped distribution of pentameric phospho-isoforms, which suggests that dephosphorylation is strongly positive cooperative so that the removal of a phosphate group enhances removal of the next (cf. Figure S12B) (Li et al., 1990). Although the data from Li et al. (1990) did not allow further characterization in terms of identifying kinetic constants for individual steps, it is likely that each dephosphorylation step increases the catalytic rate constant or the substrate affinity (or both) for the subsequent step. To account for the cooperativity observed in PLN pentamer dephosphorylation (Li et al., 1990), we assumed that PP1 (since it is the phosphatase responsible for most PLN dephosphorylation (MacDougall et al., 1991; Steenaart et al., 1992)) dephosphorylates pentamers by a positive cooperative mechanism and introduced two dimensionless control parameters φ≤1 and χ≥1. For pentamers with n≥1 phosphorylated subunits, we multiplied $k_{cat,PP1:PLN_{5}}$ with factor $\varphi^{n-1}$ and $K_{m,PP1:PLN_{5}}$ with factor $\chi^{n-1}$. For φ<1 we thus increase the turnover number for each phosphate group removed from a pentamer, with $k_{cat,PP1:PLN_{5}}$ being an upper limit. For χ>1 we thus reduce the Michaelis-constant for each phosphate group removed from a pentamer, with $K_{m,PP1:PLN_{5}}$ representing highest substrate affinity (lowest $K_{m}$ value). Such modes of cooperativity can be called *v-type* and *k-type* cooperativity, respectively (Fersht, 1999) (see Figure S12C for an illustration). Since v-type cooperativity is the simpler assumption (as it does not influence the competition terms determined by the $K_{m_{j}}$ values in the rate laws), the default parameter set assumes only presence of v-type cooperativity. Where the influence of k-type cooperativity is studied, deviations from this default assumption are explicitly mentioned.

For simplicity and lack of data suggesting otherwise, we assume $K_{m,PP1:PLN_{1}}=K_{m,PP1:PLN_{5}}$ and $k_{cat,PP1:PLN_{1}}=k_{cat,PP1:PLN_{5}}$, implying that the kinetic constants of monomer dephosphorylation pose an upper efficiency limit for pentamer dephosphorylation. If one assumes that these pose a lower limit for pentamer dephosphorylation, simulated pentamer phosphorylation occurs slower and at lower steady-state levels than monomers (data not shown), contradicting experimental observations (Wittmann et al., 2015). Potentially, this could be amended by assuming higher $k_{cat}$ and lower $K_{m}$ values for pentamer phosphorylation by PKA compared to monomers. However, since available kinetic data is not sufficient to distinguish between these possibilities, our current implementation of cooperative pentamer dephosphorylation is one of the simplest and requires the fewest assumptions.

Following the outlined rationale of the chosen rate laws, the complete list of the reaction rates of the model is given by:

##### Oligomer association/dissociation rates

$$v1=k_{1}\cdot {\left[PLN_{1}^{\left(p0\right)}\right]}^{2},\;v2=k_{2}\cdot \left[PLN_{2}^{\left(p0\right)}\right],\;v3=2\cdot k_{1}\cdot \left[PLN_{1}^{\left(p0\right)}\right]\cdot \left[PLN_{1}^{\left(p1\right)}\right],$$

$$v4=k_{2}\cdot \left[PLN_{2}^{\left(p1\right)}\right],\;v5=k_{1}\cdot \omega \cdot {\left[PLN_{1}^{\left(p1\right)}\right]}^{2},\;v6=k_{2}\cdot \left[PLN_{2}^{\left(p2\right)}\right],$$

$$v7=k_{3}\cdot {\left[PLN_{2}^{\left(p0\right)}\right]}^{2},\;v8=k_{4}\cdot \left[PLN_{4}^{\left(p0\right)}\right],\;v9=2\cdot k_{3}\cdot \left[PLN_{2}^{\left(p0\right)}\right]\cdot \left[PLN_{2}^{\left(p1\right)}\right],$$

$$v10=k_{4}\cdot \left[PLN_{4}^{\left(p1\right)}\right],\;v11=2\cdot k_{3}\cdot \left[PLN_{2}^{\left(p0\right)}\right]\cdot \left[PLN_{2}^{\left(p2\right)}\right],\;v12=\frac{1}{2}\cdot k_{4}\cdot \left[PLN_{4}^{\left(p2\right)}\right],$$

$$v13=k_{3}\cdot \omega \cdot {\left[PLN_{2}^{\left(p1\right)}\right]}^{2},\;v14=\frac{1}{2}\cdot k_{4}\cdot \left[PLN_{4}^{\left(p2\right)}\right],\;v15=2\cdot k_{3}\cdot \omega^{2}\cdot \left[PLN_{2}^{\left(p1\right)}\right]\cdot \left[PLN_{2}^{\left(p2\right)}\right],$$

$$v16=k_{4}\cdot \left[PLN_{4}^{\left(p3\right)}\right],\;v17=k_{3}\cdot \omega^{3}\cdot {\left[PLN_{2}^{\left(p2\right)}\right]}^{2},\;v18=k_{4}\cdot \left[PLN_{4}^{\left(p4\right)}\right],\;v19=k_{5}\cdot \left[PLN_{1}^{\left(p0\right)}\right]\cdot \left[PLN_{4}^{\left(p0\right)}\right],$$

$$v20=k_{6}\cdot \left[PLN_{5}^{\left(p0\right)}\right],\;v\;21=k_{5}\cdot \left[PLN_{1}^{\left(p1\right)}\right]\cdot \left[PLN_{4}^{\left(p0\right)}\right],\;v22=\frac{1}{2}\cdot k_{6}\cdot \left[PLN_{5}^{\left(p1\right)}\right],$$

$$v23=k_{5}\cdot \left[PLN_{1}^{\left(p0\right)}\right]\cdot \left[PLN_{4}^{\left(p1\right)}\right],\;v24=\frac{1}{2}\cdot k_{6}\cdot \left[PLN_{5}^{\left(p1\right)}\right],\;v25=k_{5}\cdot \omega \cdot \left[PLN_{1}^{\left(p1\right)}\right]\cdot \left[PLN_{4}^{\left(p1\right)}\right],$$

$$v26=\frac{1}{2}\cdot k_{6}\cdot \left[PLN_{5}^{\left(p2\right)}\right],\;v27=k_{5}\cdot \left[PLN_{1}^{\left(p0\right)}\right]\cdot \left[PLN_{4}^{\left(p2\right)}\right],\;v28=\frac{1}{2}\cdot k_{6}\cdot \left[PLN_{5}^{\left(p2\right)}\right],$$

$$v29=k_{5}\cdot \omega^{2}\cdot \left[PLN_{1}^{\left(p1\right)}\right]\cdot \left[PLN_{4}^{\left(p2\right)}\right],\;v30=\frac{1}{2}\cdot k_{6}\cdot \left[PLN_{5}^{\left(p3\right)}\right],\;v31=k_{5}\cdot \left[PLN_{1}^{\left(p0\right)}\right]\cdot \left[PLN_{4}^{\left(p3\right)}\right],$$

$$v32=\frac{1}{2}\cdot k_{6}\cdot \left[PLN_{5}^{\left(p3\right)}\right],\;v33=k_{5}\cdot \omega^{3}\cdot \left[PLN_{1}^{\left(p1\right)}\right]\cdot \left[PLN_{4}^{\left(p3\right)}\right],\;v34=\frac{1}{2}\cdot k_{6}\cdot \left[PLN_{5}^{\left(p4\right)}\right],$$

$$v35=k_{5}\cdot \left[PLN_{1}^{\left(p0\right)}\right]\cdot \left[PLN_{4}^{\left(p4\right)}\right],\;v36=\frac{1}{2}\cdot k_{6}\cdot \left[PLN_{5}^{\left(p4\right)}\right],\;v37=k_{5}\cdot \omega^{4}\cdot \left[PLN_{1}^{\left(p1\right)}\right]\cdot \left[PLN_{4}^{\left(p4\right)}\right],$$

$$v38=k_{6}\cdot \left[PLN_{5}^{\left(p5\right)}\right].$$

##### Phosphorylation and dephosphorylation of PLN

$$v39=\frac{\left[PKA\right]k_{cat,PKA:PLN_{1}}\left[PLN_{1}^{\left(p0\right)}\right]}{K_{m,PKA:PLN_{1}}\left(1+\sum_{i\in I}\left(\frac{\left[PLN_{5}^{\left(pi\right)}\right]}{K_{m,PKA:PLN_{5}}}\right)+\frac{\left[Inh-1^{\left(p0\right)}\right]}{K_{m,PKA:Inh-1}}\right)+\left[PLN_{1}^{\left(p0\right)}\right]}$$

$$v40=\frac{\left[PP1\right]k_{cat,PP1:PLN_{1}}\left[PLN_{1}^{\left(p1\right)}\right]}{K_{m,PP1:PLN_{1}}\left(1+\sum_{j\in J}\frac{\left[PLN_{5}^{\left(pj\right)}\right]}{\chi^{j-1}K_{m,PP1:PLN_{5}}}\right)+\left[PLN_{1}^{\left(p1\right)}\right]}$$

$$v41=\frac{V_{max,PP2A:PLN_{1}}\left[PLN_{1}^{\left(p1\right)}\right]}{K_{m,PP2A}+\left[PLN_{1}^{\left(p1\right)}\right]+\sum_{j\in J}\left(\left[PLN_{5}^{\left(pj\right)}\right]\right)+\left[Inh-1^{\left(p1\right)}\right]}$$

$$v42=\frac{\left[PKA\right]k_{cat,PKA:PLN_{5}}\left[PLN_{5}^{\left(p0\right)}\right]}{K_{m,PKA:PLN_{5}}\left(1+\frac{\left[PLN_{1}^{\left(p0\right)}\right]}{K_{m,PKA:PLN_{1}}}+\sum_{i\in I\setminus \left\{0\right\}}\left(\frac{\left[PLN_{5}^{\left(pi\right)}\right]}{K_{m,PKA:PLN_{5}}}\right)+\frac{\left[Inh-1^{\left(p0\right)}\right]}{K_{m,PKA:Inh-1}}\right)+\left[PLN_{5}^{\left(p0\right)}\right]}$$

$$v43=\frac{\left[PP1\right]k_{cat,PP1:PLN_{5}}\left[PLN_{5}^{\left(p1\right)}\right]}{K_{m,PP1:PLN_{5}}\left(1+\frac{\left[PLN_{1}^{\left(p1\right)}\right]}{K_{m,PP1:PLN_{1}}}+\sum_{j\in J\setminus \left\{1\right\}}\frac{\left[PLN_{5}^{\left(pj\right)}\right]}{\chi^{j-1}K_{m,PP1:PLN_{5}}}\right)+\left[PLN_{5}^{\left(p1\right)}\right]}$$

$$v44=\frac{V_{max,PP2A:PLN_{5}}\left[PLN_{5}^{\left(p1\right)}\right]}{K_{m,PP2A}+\left[PLN_{1}^{\left(p1\right)}\right]+\sum_{j\in J}\left(\left[PLN_{5}^{\left(pj\right)}\right]\right)+\left[Inh-1^{\left(p1\right)}\right]}$$

$$v45=\frac{\left[PKA\right]k_{cat,PKA:PLN_{5}}\left[PLN_{5}^{\left(p1\right)}\right]}{K_{m,PKA:PLN_{5}}\left(1+\frac{\left[PLN_{1}^{\left(p0\right)}\right]}{K_{m,PKA:PLN_{1}}}+\sum_{i\in I\setminus \left\{1\right\}}\left(\frac{\left[PLN_{5}^{\left(pi\right)}\right]}{K_{m,PKA:PLN_{5}}}\right)+\frac{\left[Inh-1^{\left(p0\right)}\right]}{K_{m,PKA:Inh-1}}\right)+\left[PLN_{5}^{\left(p1\right)}\right]}$$

$$v46=\frac{\varphi \left[PP1\right]k_{cat,PP1:PLN_{5}}\left[PLN_{5}^{\left(p2\right)}\right]}{\chi K_{m,PP1:PLN_{5}}\left(1+\frac{\left[PLN_{1}^{\left(p1\right)}\right]}{K_{m,PP1:PLN_{1}}}+\sum_{j\in J\setminus \left\{2\right\}}\frac{\left[PLN_{5}^{\left(pj\right)}\right]}{\chi^{j-1}K_{m,PP1:PLN_{5}}}\right)+\left[PLN_{5}^{\left(p2\right)}\right]}$$

$$v47=\frac{V_{max,PP2A:PLN_{5}}\left[PLN_{5}^{\left(p2\right)}\right]}{K_{m,PP2A}+\left[PLN_{1}^{\left(p1\right)}\right]+\sum_{j\in J}\left(\left[PLN_{5}^{\left(pj\right)}\right]\right)+\left[Inh-1^{\left(p1\right)}\right]}$$

$$v48=\frac{\left[PKA\right]k_{cat,PKA:PLN_{5}}\left[PLN_{5}^{\left(p2\right)}\right]}{K_{m,PKA:PLN_{5}}\left(1+\frac{\left[PLN_{1}^{\left(p0\right)}\right]}{K_{m,PKA:PLN_{1}}}+\sum_{i\in I\setminus \left\{2\right\}}\left(\frac{\left[PLN_{5}^{\left(pi\right)}\right]}{K_{m,PKA:PLN_{5}}}\right)+\frac{\left[Inh-1^{\left(p0\right)}\right]}{K_{m,PKA:Inh-1}}\right)+\left[PLN_{5}^{\left(p2\right)}\right]}$$

$$v49=\frac{\varphi^{2}\left[PP1\right]k_{cat,PP1:PLN_{5}}\left[PLN_{5}^{\left(p3\right)}\right]}{\chi^{2}K_{m,PP1:PLN_{5}}\left(1+\frac{\left[PLN_{1}^{\left(p1\right)}\right]}{K_{m,PP1:PLN_{1}}}+\sum_{j\in J\setminus \left\{3\right\}}\frac{\left[PLN_{5}^{\left(pj\right)}\right]}{\chi^{j-1}K_{m,PP1:PLN_{5}}}\right)+\left[PLN_{5}^{\left(p3\right)}\right]}$$

$$v50=\frac{V_{max,PP2A:PLN_{5}}\left[PLN_{5}^{\left(p3\right)}\right]}{K_{m,PP2A}+\left[PLN_{1}^{\left(p1\right)}\right]+\sum_{j\in J}\left(\left[PLN_{5}^{\left(pj\right)}\right]\right)+\left[Inh-1^{\left(p1\right)}\right]}$$

$$v51=\frac{\left[PKA\right]k_{cat,PKA:PLN_{5}}\left[PLN_{5}^{\left(p3\right)}\right]}{K_{m,PKA:PLN_{5}}\left(1+\frac{\left[PLN_{1}^{\left(p0\right)}\right]}{K_{m,PKA:PLN_{1}}}+\sum_{i\in I\setminus \left\{3\right\}}\left(\frac{\left[PLN_{5}^{\left(pi\right)}\right]}{K_{m,PKA:PLN_{5}}}\right)+\frac{\left[Inh-1^{\left(p0\right)}\right]}{K_{m,PKA:Inh-1}}\right)+\left[PLN_{5}^{\left(p3\right)}\right]}$$

$$v52=\frac{\varphi^{3}\left[PP1\right]k_{cat,PP1:PLN_{5}}\left[PLN_{5}^{\left(p4\right)}\right]}{\chi^{3}K_{m,PP1:PLN_{5}}\left(1+\frac{\left[PLN_{1}^{\left(p1\right)}\right]}{K_{m,PP1:PLN_{1}}}+\sum_{j\in J\setminus \left\{4\right\}}\frac{\left[PLN_{5}^{\left(pj\right)}\right]}{\chi^{j-1}K_{m,PP1:PLN_{5}}}\right)+\left[PLN_{5}^{\left(p4\right)}\right]}$$

$$v53=\frac{V_{max,PP2A:PLN_{5}}\left[PLN_{5}^{\left(p4\right)}\right]}{K_{m,PP2A}+\left[PLN_{1}^{\left(p1\right)}\right]+\sum_{j\in J}\left(\left[PLN_{5}^{\left(pj\right)}\right]\right)+\left[Inh-1^{\left(p1\right)}\right]}$$

$$v54=\frac{\left[PKA\right]k_{cat,PKA:PLN_{5}}\left[PLN_{5}^{\left(p4\right)}\right]}{K_{m,PKA:PLN_{5}}\left(1+\frac{\left[PLN_{1}^{\left(p0\right)}\right]}{K_{m,PKA:PLN_{1}}}+\sum_{i\in I\setminus \left\{4\right\}}\left(\frac{\left[PLN_{5}^{\left(pi\right)}\right]}{K_{m,PKA:PLN_{5}}}\right)+\frac{\left[Inh-1^{\left(p0\right)}\right]}{K_{m,PKA:Inh-1}}\right)+\left[PLN_{5}^{\left(p4\right)}\right]}$$

$$v55=\frac{\varphi^{4}\left[PP1\right]k_{cat,PP1:PLN_{5}}\left[PLN_{5}^{\left(p5\right)}\right]}{\chi^{4}K_{m,PP1:PLN_{5}}\left(1+\frac{\left[PLN_{1}^{\left(p1\right)}\right]}{K_{m,PP1:PLN_{1}}}+\sum_{j\in J\setminus \left\{5\right\}}\frac{\left[PLN_{5}^{\left(pj\right)}\right]}{\chi^{j-1}K_{m,PP1:PLN_{5}}}\right)+\left[PLN_{5}^{\left(p5\right)}\right]}$$

$$v56=\frac{V_{max,PP2A:PLN_{5}}\left[PLN_{5}^{\left(p5\right)}\right]}{K_{m,PP2A}+\left[PLN_{1}^{\left(p1\right)}\right]+\sum_{j\in J}\left(\left[PLN_{5}^{\left(pj\right)}\right]\right)+\left[Inh-1^{\left(p1\right)}\right]}$$

##### Reactions involving inhibitor-1

$$v57=\frac{\left[PKA\right]k_{cat,PKA:Inh-1}\left[Inh-1^{\left(p0\right)}\right]}{K_{m,PKA:Inh-1}\left(1+\sum_{i\in I}\left(\frac{\left[PLN_{5}^{\left(pi\right)}\right]}{K_{m,PKA:PLN_{5}}}\right)+\frac{\left[PLN_{1}^{\left(p0\right)}\right]}{K_{m,PKA:PLN_{1}}}\right)+\left[Inh-1^{\left(p0\right)}\right]}$$

$$v58=\frac{V_{max,PP2A:Inh-1}\left[Inh-1^{\left(p1\right)}\right]}{K_{m,PP2A}+\left[PLN_{1}^{\left(p1\right)}\right]+\sum_{j\in J}\left(\left[PLN_{5}^{\left(pj\right)}\right]\right)+\left[Inh-1^{\left(p1\right)}\right]}$$

$$v59=k_{7}\left[Inh-1^{\left(p1\right)}\right]\left[PP1\right]$$

$$v60=k_{8}\left[PP1:Inh-1^{\left(p1\right)}\right]$$

##### Model parameters and initial conditions

A mathematical model needs parameters and initial conditions to make useful predictions. In order to obtain realistic parameters values and protein concentrations we searched the literature and previously published models related to calcium handling and β-adrenergic signaling in cardiomyocytes, as well as phospholamban, PKA, PP1 and inhibitor-1.

**Table:** Table: review protein concentrations

Protein concentration	Value	Source	Comments
${\left[PLN\right]}_{tot}$	≈ 250 μM	this study	
	106 μM	(Saucerman et al., 2003)	
	> 50 μM	(Bers, 2002; Rigatti et al., 2015)	
	38 μM	(Soltis and Saucerman 2010)	
[PKA]	0.59 μM	(Saucerman et al., 2003)	
	0.5176 μM	(Bondarenko, 2014)	
	0.48 μM	(Saucerman et al., 2004)	
${\left[PP1\right]}_{tot}$	0.89 μM	(Saucerman et al., 2003, Saucerman et al., 2004)	
	0.2 μM	(Bondarenko, 2014)	
	0.5 μM∗	(Legewie et al., 2008)	(skeletal muscle)
${\left[Inh-1\right]}_{tot}$	0.3 μM	(Saucerman et al., 2003)	
	0.08543 μM	(Bondarenko, 2014)	

∗ Calculated by the number of protein molecules per cell given in the reference and the rule of thumb that in an “average” eukaryotic cell, 1000 molecules of a protein roughly correspond to a cellular concentration of 1 nM (Luby-Phelps, 2000; BioNumbers BNID 104519).

**Table:** Table: review kinetic and equilibrium constants

Parameter	Value	Source	Comments
$k_{cat,PKA:PLN}$	54 s^-1^	(Saucerman et al., 2003)	($k_{cat}$ for PLN phosphorylation by PKA)
	21 s^-1^	(Rigatti et al., 2015)	
	23.4 s-^1^	(Ha et al., 2011)	${\text{PLN}}_{1-20}$, no lipid environment
	22.3 – 25 s^-1^	(Masterson et al., 2011)	${\text{PLN}}_{1-19}$, different environments
	13 – 21.9 s-^1^	(Masterson et al., 2011)	AFA-PLN, different environments
$K_{m,PKA:PLN}$	21 μM	(Saucerman et al., 2003)	($K_{m}$ for PLN phosphorylation by PKA)
	12.5 μM	(Rigatti et al., 2015)	
	93.3 μM	(Ha et al., 2011)	PLN_1-20_, no lipid environment
	36.4 – 90.1 μM	(Masterson et al., 2011)	PLN_1-19_, different environments
	47.6 – 238.1∗μM	(Masterson et al., 2011)	AFA-PLN, different environments
$k_{cat,PP1:PLN}$	8.5 s^-1^	(Saucerman et al., 2003)	($k_{cat}$ for PLN phosphorylation by PKA)
$K_{m,PP1:PLN}$	7 μM	(Saucerman et al., 2003)	($K_{m}$ for PLN dephosphorylation by PP1)
$V_{max,PP2A:PLN}$	0.708 μM s^-1^	(MacDougall et al., 1991)	guesstimate^#^, ($V_{max}$ for PLN dephosphorylation by PP2A)
$V_{max,PP2A:Inh-1}$	14 μM s^-1^	(Saucerman et al., 2003)	($V_{max}$ for inhibitor-1 dephosphorylation by PP2A)
$k_{cat,PKA:Inh-1}$	60 s^-1^	(Saucerman et al., 2003)	($k_{cat}$ for inhibitor-1 phosphorylation by PKA)
$K_{m,PKA:Inh-1}$	1 μM	(Saucerman et al., 2003)	($K_{m}$ for inhibitor-1 phosphorylation by PKA)
$K_{m,PP2A}$	1 μM	(Saucerman et al., 2003)	($K_{m}$ for inhibitor-1 dephosphorylation by PP2A)
$K_{d,PP1:Inh-1^{\left(p1\right)}}$	1 nM	(Saucerman et al., 2003)	($K_{d}$ for complex of PP1 and phosphorylated inhibitor-1)

∗ Not included in calculation of median value for default parameter set. #MacDougall et al. (1991) reported that PP1 accounts for 70-90% and PP2A for the remaining dephosphorylation activity toward phospholamban (and to a small extend PP2C, too). We thus assumed that PP2A dephosphorylation accounts for approximately 20% of the total dephosphorylation activity toward phospholamban observed by MacDougall et al. (1991). We furthermore assumed the same $K_{m}$ value as for inhibitor-1 dephosphorylation.

##### Parameter ω (dynamic equilibrium of PLN)

Since individual oligomerization steps and the influence of phosphorylation on their rate constants have not been studied before, we used our own data and those from Hou et al. (2008) to obtain a first estimate for ω as described in the subsection ‘Parameter estimation and model selection’ below.

**Table:** 

Parameter	Value	Source	Comments
ω	≈1.066	this study	based on data from Figure S8E
	≈1.044	(Hou et al., 2008)	see subsection ‘Parameter estimation and model selection’

It is important to note, however, that the extend to which this effect can be observed varies considerably. Wittmann et al. (2015), for instance, observed no significant increase in pentamerization upon Ser16 phosphorylation in forskolin stimulated HEK293 cells. Cornea et al. (1997), in contrast, found a near complete pentamerization of Ser16-phosphorylated, recombinant PLN in DOPC lipid bilayers using electron paramagnetic resonance spectroscopy. The effect observed by Hou et al. (2008), could be an underestimation given that phospho-mimetic mutations are not always faithful experimental models for real phosphorylation (Kampourakis et al., 2018). Moreover, the lipid environment, too, plays an important role for both pentamerization (Zhang et al., 2005) and the dynamic equilibrium effect (this study). Taken together, these considerations make it difficult to get a precise estimate from the data so far. We thus decided to opt for a ‘guesstimate’ of ω=1.25, which is higher than the estimates based on our or Hou et al. (2008)’s data, but still lower than what one would expect for the near complete pentamerization observed by Cornea et al. (1997).

##### Cooperative dephosphorylation of pentamers

To the best of our knowledge, no kinetic parameters for the (de-)phosphorylation of pentameric phospholamban have been determined so far. In the absence of better evidence we mostly assumed the parameters to be the same as for monomeric phospholamban. An exception to this is the dephosphorylation of pentameric PLN by PP1. In order to implement the positive cooperativity of dephosphorylation reported by (Li et al., 1990), we assumed a pronounced v-type cooperativity of φ=0.2 and the absence of k-type cooperativity, i.e., χ=1.

##### Set of default parameters and initial conditions

Based on these reviews and considerations we composed a default parameter set, which was used for all simulations and analyses unless specified otherwise in the simulation protocols given below. Oligomerization parameter values are from the best fit parameter set of mass action model 2 calibrated with our experimental data (see subsection ‘A mass action kinetics model of PLN pentamer assembly’).

**Table:** Table: default parameter values and initial conditions

Parameter/IC	Value	Comments
${\left[PLN\right]}_{tot}$	250 μM	
[PKA]	0.59 μM	
${\left[PP1\right]}_{tot}$	0.89 μM	free $\left[PP1\right]\left(t=0\right)={\left[PP1\right]}_{tot}$ unless stated otherwise
${\left[Inh-1\right]}_{tot}$	0.3 μM	$\left[Inh-1^{\left(p0\right)}\right]\left(t=0\right)={\left[Inh-1\right]}_{tot}$ unless stated otherwise
$k_{1}$	144724 mol s^-1^	(rate constant for dimer formation; best fit parameter set)
$k_{2}$	5518.45 s^-1^	(rate constant for dimer dissociation; best fit parameter set)
$k_{3}$	72672.8 mol s^-1^	(rate constant for tetramer formation; best fit parameter set)
$k_{4}$	1.20089 s^-1^	(rate constant for tetramer dissociation; best fit parameter set)
$k_{5}$	1.83 $\times 10^{8}$ mol s^-^^1^	(rate constant for pentamer formation; best fit parameter set)
$k_{6}$	0.13275 s^-1^	(rate constant for pentamer dissociation; best fit parameter set)
$k_{7}$	5$\times 10^{4}$ mol s^-1^	(association rate constant for binding of phosphorylated inhibitor-1
		to PP1; matched with $k_{8}$ to a $K_{d}$ of 1 nM)
$k_{8}$	5$\times 10^{-5}$ s^-^^1^	(dissociation rate constant of complex between phosphorylated
		inhibitor-1 and PP1; matched with $k_{7}$ to a $K_{d}$ of 1 nM)
ω	1.25	(increased oligomerization upon PLN phosphorylation)
$k_{cat,PKA:PLN_{1}}$	23 s^-1^	($k_{cat}$ for PLN_1_ phosphorylation by PKA; median of reviewed values)
$K_{m,PKA:PLN_{1}}$	42 μM	($K_{m}$ for PLN_1_ phosphorylation by PKA; median of reviewed values)
$k_{cat,PKA:PLN_{5}}$	23 s^-1^	($k_{cat}$ for PLN_5_ phosphorylation by PKA; assumed equal to monomers)
$K_{m,PKA:PLN_{5}}$	42 μM	($K_{m}$ for PLN_5_ phosphorylation by PKA; assumed equal to monomers)
$k_{cat,PP1:PLN_{1}}$	8.5 s^-^^1^	($k_{cat}$ for PLN_1_ dephosphorylation by PP1)
$K_{m,PP1:PLN_{1}}$	7 μM	($K_{m}$ for PLN_1_ dephosphorylation by PP1)
$k_{cat,PP1:PLN_{5}}$	8.5 s^-^^1^	(baseline $k_{cat}$ for PLN_5_ dephosphorylation by PP1)
$K_{m,PP1:PLN_{5}}$	7 μM	(baseline $K_{m}$ for PLN_5_ dephosphorylation by PP1)
$V_{max,PP2A:PLN}$	0.708 μM s^-1^	($V_{max}$ for PLN dephosphorylation by PP2A)
$V_{max,PP2A:Inh-1}$	14 μM s^-1^	($V_{max}$ for inhibitor-1 dephosphorylation by PP2A)
$k_{cat,PKA:Inh-1}$	60 s^-1^	($k_{cat}$ for inhibitor-1 phosphorylation by PKA)
$K_{m,PKA:Inh-1}$	1 μM	($K_{m}$ for inhibitor-1 phosphorylation by PKA)
$K_{m,PP2A}$	1 μM	($K_{m}$ for inhibitor-1 and PLN dephosphorylation by PP2A)
$K_{d,PP1:Inh-1^{\left(p1\right)}}$	1 nM	($K_{d}$ for complex of PP1 and phosphorylated inhibitor-1)
φ	0.2	(v-type cooperativity for PLN_2_ dephosphorylation by PP1)
χ	1	(k-type cooperativity for PLN_5_ dephosphorylation by PP1)

#### Computational procedures

##### Model implementation and software

All models were implemented as MATLAB® (v2019b) scripts for numerical simulation and analysis. Simulations were performed with the ode23s integrator on an Asus® laptop PC with Intel® Corei7-7500U CPU @ 2.70GHz, 2904 Mhz, 2 Core(s) processor, 8GB RAM running under Microsoft® Windows 10 OS. Simulation protocols describing the used parameter values and initial conditions for each simulation shown in the figures of the main text (where deviating from the default parameter set) can be found further below.

##### Parameter estimation and model selection

Parameter estimation for association and dissociation rate constants of the oligomerization reactions in our mass-action kinetics models was performed in COPASI (v4.26) using the genetic algorithm with 250 generations and a population size of 40. Parameters were constrained by the detailed balance relationships described in subsection ‘A mass action kinetics model of PLN pentamer assembly’. Furthermore, association rate constants were constrained to lie between $10^{3}$mol⋅s^-1^ and $10^{9}$mol⋅s^-1^, whereas dissociation constants were constrained to lie between $10^{-5}$s^-1^ and $10^{6}$s^-1^. For each model variant, 30 independent parameter estimation runs with randomized initial values parameters were performed. For model selection, the AIC was calculated as described in Flöttmann et al. (2008) by $AIC=2\cdot k+n\cdot \left(ln\left(\frac{SSE}{n}\right)+1\right)$, where *k* is the number of model parameters, *n* is the number of experimental observations and SSE is the sum of weighted squared errors for the best fit parameter set of a model. The AIC score balances how well a model fits the data versus model complexity in number of parameter values and penalizes deviations from experimental data and high number of model parameters. The lower the AIC score, the better. If two models fit the data equally well, the model with fewer parameters is rewarded a lower AIC score because it has less unnecessary complexity.

Estimation of parameter ω to our own data was performed by assuming that the increased pentamerization shown in Figure S8E reflects the steady-state situation and by using the MATLAB® function nlinfit to fit the parameter to this data.

Calculation of parameter ω based on the data from Hou et al. (2008) was done as follows. The apparent monomer-pentamer equilibrium in a monomer-dimer-tetramer-pentamer model such as ours can be characterized by the apparent dissociation constant:

$$K_{d\;1,5}^{app}=\frac{{\left[PLN_{1}\right]}^{5}}{\left[PLN_{5}\right]}.$$

Since we have

$$\left[PLN_{5}\right]=K_{4,5}\left[PLN_{4}\right]\left[PLN_{1}\right]$$

$$=K_{2,4}K_{4,5}{\left[PLN_{2}\right]}^{2}\left[PLN_{1}\right]$$

$$=K_{1,2}^{2}K_{2,4}K_{4,5}{\left[PLN_{1}\right]}^{5},$$

we can write

$$K_{d\;1,5}^{app}=\frac{{\left[PLN_{1}\right]}^{5}}{\left[PLN_{5}\right]}=\frac{1}{K_{1,2}^{2}K_{2,4}K_{4,5}}=\frac{k_{2}^{2}k_{4}k_{6}}{k_{1}^{2}k_{3}k_{5}},$$

where in the last step the equilibrium constants were substituted with their constitute rate constants. For completely phosphorylated PLN, the derivation of the apparent dissociation constant is identical except for factoring in ω into the individual equilibria:

$$K_{d\;1,5}^{app,pPLN}=\frac{{\left[PLN_{1}^{\left(p1\right)}\right]}^{5}}{\left[PLN_{5}^{\left(p5\right)}\right]}={\left(\frac{k_{2}}{\omega k_{1}}\right)}^{2}\left(\frac{k_{4}}{\omega^{3}k_{3}}\right)\left(\frac{k_{6}}{\omega^{4}k_{5}}\right)=\frac{1}{\omega^{9}}\frac{k_{2}^{2}k_{4}k_{6}}{k_{1}^{2}k_{3}k_{5}}.$$

Using the apparent $K_{d}$ values from Hou et al. (2008) (given in arbitrary units) for Ser16A PLN (unphosphorylated) and Ser16E PLN (mimicking phosphorylated PLN) we arrive at:

$$\frac{K_{d\;1,5}^{app}}{K_{d\;1,5}^{app,pPLN}}=\frac{2.2}{1.5}=\frac{\frac{k_{2}^{2}k_{4}k_{6}}{k_{1}^{2}k_{3}k_{5}}}{\frac{1}{\omega^{9}}\frac{k_{2}^{2}k_{4}k_{6}}{k_{1}^{2}k_{3}k_{5}}}=\omega^{9}\Leftrightarrow \omega =\sqrt[{9}]{\frac{2.2}{1.5}}\approx 1.044$$

##### Bifurcation diagrams

Bifurcation diagrams were generated using a custom algorithm described previously (Koch, 2020) to make the implementation of bifurcation analysis into the simulation/analysis pipeline easier (e.g., for sensitivity analysis of the bistable range). The algorithm iteratively identifies/approximates the unstable steady states of bistable systems numerically, but does not detect other types of bifurcations (e.g., Hopf-bifurcations). For more details, please refer to: https://www.ebi.ac.uk/biomodels/MODEL1910220002.

##### Local sensitivity analysis

Local sensitivity analyses was performed as described in Ingalls (2013). We analyzed two steady states which were chosen to be sufficiently remote from the saddle node bifurcations SN1 and SN2 of the model: a low relative phosphorylation level ([PKA] = 0.13 μM), and a high relative phosphorylation level ([PKA] = 0.25 M). Relative sensitivities of (relative) PLN monomer phosphorylation at steady state upon perturbing the nominal value of each parameter *p* by dp=1% at a time were calculated as $s_{r}\left(p\right)=\frac{dPLN_{1}^{\left(rp\right)}/PLN_{1}^{\left(rp\right)}}{dp/p}$, where $rPLN_{1}^{\left(p1\right)}=\frac{\left[PLN_{1}^{\left(p1\right)}\right]}{\left[PLN_{1}^{\left(p0\right)}\right]+\left[PLN_{1}^{\left(p1\right)}\right]}$.

For bifurcation sensitivity analysis, relative sensitivities were determined in a similar fashion, but instead of focusing on the steady state concentration of relative PLN monomer phosphorylation, relative sensitivities of the bistable range (|SN1-SN2|) were calculated. In order to obtain well detectable changes in relative sensitivities with a limited granularity of the bifurcation algorithm, a perturbation of dp=10% was chosen.

##### Probing the multi-dimensional parameter space for its influence on bistability

In order to probe how bistability depends on the multi-dimensional parameter space of our model, we decided to perform an analysis developed by Nguyen et al. (2015). First, we used the review of parameter values and protein concentrations to guide the construction of ranges of physiologically plausible values in which the true values are likely to lie. As we could not get our model to be executed by the python implementation provided in Nguyen et al. (2015), we decided to implement the analysis in the MATLAB® code of our model. However, as our model equations are quite stiff for some combinations of parameters, we first performed a model reduction and replaced one ODE for an algebraic expression in order to speed up the numerical solution of the model. Due to ease of implementation, a quasi-steady-state (QSS) approximation of PP1 is particularly suited to replace an ODE with an algebraic expression. Although this can affect the model dynamics (and thereby eliminate e.g., the response delay of the inhibitor-1 FFL), we used the reduced model only for steady state analyses for which the QSS assumption is (trivially) fulfilled (Ingalls, 2013). At steady state we find that:

$$\frac{d}{dt}\left[PP1\right]\left(t\right)=k_{8}\left[Inh-1^{\left(p1\right)}:PP1\right]\left(t\right)-k_{7}\left[Inh-1^{\left(p1\right)}\right]\left(t\right)\left[PP1\right]\left(t\right)=0$$

Assuming that PP1 is quasi at steady state compared to the other processes in the reaction network, we denote its concentration in the reduced model by $\left[P\tilde{P}1\right]$. From the ODEs, we can identify the following conservation laws:

$${\left[PP1\right]}_{tot}=\left[PP1\right]\left(t\right)+\left[PP1:Inh-1^{\left(p1\right)}\right]\left(t\right)$$

$${\left[Inh-1\right]}_{tot}=\left[Inh-1^{\left(p0\right)}\right]\left(t\right)+\left[Inh-1^{\left(p1\right)}\right]\left(t\right)+\left[PP1:Inh-1^{\left(p1\right)}\right]\left(t\right)$$

Applying these to the first equation and solving the resulting quadratic equation for $\tilde{\left[PP1\right]}$ using the pq-formula gives (for physical reasons only the positive solution is given):

$$\left[P\tilde{P}1\right]\left(t\right)=-\left(\frac{k_{8}+k_{7}\left({\left[Inh-1\right]}_{tot}-\left[Inh-1^{\left(p0\right)}\right]\left(t\right)-{\left[PP1\right]}_{tot}\right)}{2k_{7}}\right)$$

$$+\sqrt{\frac{\left(k_{8}+k_{7}{\left({\left[Inh-1\right]}_{tot}-\left[Inh-1^{\left(p0\right)}\right]\left(t\right)-{\left[PP1\right]}_{tot}\right)}^{2}\right)}{4k_{7}^{2}}-\frac{k_{8}}{k_{7}}{\left[PP1\right]}_{tot}}$$

Having eliminated the ODE for PP1 with this algebraic expression, we observed no deviations in the steady state behavior but a marked performance gain.

For the intended analysis, parameters and initial conditions were sampled randomly from a uniform distribution determined by a range of physiologically plausible values. Using the reduced model, two simulations with either completely unphosphorylated or completely phosphorylated PLN (both monomeric and pentameric) as initial conditions were performed with simulation endpoint $t_{end}=5\times 10^{5}s$. To test whether the model reached steady state, the algorithm checked whether monomer concentration was stable for at least three time points before reaching the end of the simulation. A parameter set was considered bistable if there was a relative difference in phosphorylation levels of > 10%. Parameter sets without a difference in phosphorylation levels or with a difference of < 10% were considered monostable since we reasoned that marginal differences in steady state phosphorylation might not be of physiological relevance (although technically still bistable). Parameter sets were normalized to the maximum value of the respective parameter range and visualized using parallel coordinate plots.

##### Frequency response analysis

Frequency response analysis was performed as described in Ingalls (2013). First, the system’s jacobian matrix

$$A=\frac{\partial f}{\partial x}=\left(\begin{array}{llll} \frac{\partial f_{1}}{\partial \left[PLN_{1}^{\left(p0\right)}\right]} & \frac{\partial f_{1}}{\partial \left[PLN_{2}^{\left(p0\right)}\right]} & \cdots & \frac{\partial f_{1}}{\partial \left[PP1\right]}\\ \frac{\partial f_{2}}{\partial \left[PLN_{1}^{\left(p0\right)}\right]} & \frac{\partial f_{2}}{\partial \left[PLN_{2}^{\left(p0\right)}\right]} & \cdots & \frac{\partial f_{2}}{\partial \left[PP1\right]}\\ \vdots & \vdots & \ddots & \vdots \\ \frac{\partial f_{17}}{\partial \left[PLN_{1}^{\left(p0\right)}\right]} & \frac{\partial f_{17}}{\partial \left[PLN_{2}^{\left(p0\right)}\right]} & \cdots & \frac{\partial f_{17}}{\partial \left[PP1\right]} \end{array}\right),$$

was calculated symbolically using MATLAB®, where $f_{1},\ldots ,f_{17}$ (**f** in vector notation) denotes the right hand sides of the model ODEs as a function of the model variables $\left[PLN_{1}^{\left(p0\right)}\right],\ldots ,\left[PP1\right]$ (**x** in vector notation) in the specified order. For the purpose of the frequency response analysis, the model input *u* was defined as [PKA] and the output *h* as relative monomer phosphorylation $\frac{\left[PLN_{1}^{\left(p1\right)}\right]}{\left[PLN_{1}^{\left(p0\right)}\right]+\left[PLN_{1}^{\left(p1\right)}\right]}$. Linearized input and output maps **B** and **C** were calculated as $B=\frac{\partial f}{\partial u}$ and $C=\frac{\partial h}{\partial x}$, respectively. The feed-through term *D* as $\frac{\partial h}{\partial u}$. After evaluating **A,B,C** and *D* at a nominal operating point, a linearized input-output system was created using the MATLAB® function ss2tf. The linearized input-output system was subsequently used for the evaluation of the system bandwidth and for creating Bode-diagrams using the MATLAB® functions bandwidth and bode, respectively, where the gain is defined as the ratio of the amplitude of the response to the amplitude of the input, and phase shift as the ratio of the time difference between response peaks to the period of the input oscillations given in degrees (Ingalls, 2013).

##### Simulation protocols

To enable reproducibility of our simulations in other software environments, we provide here the simulation protocols, initial conditions and parameter values underlying the presented simulations in the main text. Figure numbers refer to the main text. For specifications of the simulations from the supplementary material, please refer to the published code.

• Figure 3**A, left**: First, 250 μM [PLN]_*tot*_ (unphosphorylated) were simulated until oligomerization reached equilibrium in the absence of any other species (no inhibitor-1, no phosphatases, no PKA). Subsequently, [PKA] = 0.1 μM and a 300 s phosphorylation time course of PLN species was simulated in the absence of phosphatases and inhibitor-1. Other initial conditions and parameters as listed in Table: default parameter values and initial conditions.

• Figure 3**A, right**: PKA was set to 0 μM and final PLN species concentrations from Figure 3A (left) were used as initial conditions for PLN. Free PP1 was set 0.1 μM and a 250 s dephosphorylation time course was simulated. Inhibitor-1 was absent. PP2A and other initial conditions and parameters as listed in Table: default parameter values and initial conditions.

• Figure 3B: red and blue lines are the same data as in Figure 3A replotted as relative monomer and pentamer phosphorylation, respectively. For the simulation in the absence of pentamers (dotted purple line), the simulations described for Figure 3A were repeated with rate constants $k_{1}=k_{2}=k_{3}=k_{4}=k_{5}=k_{6}=0$ (to prevent oligomerization) and initial PLN concentration equimolar to the monomer concentration of [PLN]_*tot*_ = 250 μM at oligomerization equilibrium (≈ 58.2 μM).

• Figure 3C: As Figures 3A and 3B, but with [PKA] = 1 nM.

• Figure 4B: First, 250 μM [PLN]_*tot*_ (unphosphorylated) were simulated until oligomerization reached equilibrium in the absence of any other species (no inhibitor-1, no phosphatases, no PKA). Subsequently, PKA was set to 0.35 μM and a 350 s phosphorylation time course of PLN species was simulated the presence of phosphatases and inhibitor-1 (concentrations as listed in Table: default parameter values and initial conditions). Subsequently, PKA was set to 0 μM and dephosphorylation was simulated for 250 s. The whole simulation was performed three times with different rate constants for binding of inhibitor-1 to PP1: $k_{7}$ = $1/2\times 10^{4}$ mol s^-1^ and $k_{8}$ = $1/2\times 10^{-5}$ s^-1^, respectively. Finally, the simulation was repeated in the absence of inhibitor-1 but with PP1 reduced by the fraction of PP1 bound to inhibitor-1 at steady state from previous simulations (to ensure the same steady state is approached). Other initial conditions and parameters as listed in Table: default parameter values and initial conditions.

• Figure 4C: (e1-e3) [PLN_1_](t = 0) = [PLN]_*tot*_ = 55 μM, $k_{1}=k_{2}=k_{3}=k_{4}=k_{5}=k_{6}=0$, $V_{max,PP2A:PLN}=V_{max,PP2A:Inh-1}=0$, $k_{cat,PKA:Inh-1}$ = 0.25 s^-1^, $K_{m,PKA:Inh-1}$ = 14 μM, t_*end*_ = 2000 s. (e1) [PKA] = 10 nM, [PP1](t = 0) = [PP1]_*tot*_ = 1 μM, [Inh-1^(*p*0)^](t = 0) = [Inh-1]_*tot*_ = 10 μM. (e2) [PKA] =10 nM, [PP1](t = 0) = [PP1]_*tot*_ = 0 μM, [Inh-1^(*p*0)^](t = 0) = [Inh-1]_*tot*_ = 0 μM. (e3) [PKA] = 10 nM, [PP1](t = 0) = [PP1]_*tot*_ = 1 μM, [Inh-1^(*p*0)^](t = 0) = [Inh-1]_*tot*_ = 0 μM.

• Figure 5A: First, 250 μM [PLN]_*tot*_ (unphosphorylated) were simulated until oligomerization reached equilibrium in the absence of any other species (no inhibitor-1, no phosphatases, no PKA). Next, ten simulations with 0%–100% (in steps of 10%) of monomeric and pentameric PLN from the first step being phosphorylated at t = 0 were run for 2000s at [PKA] = 0.21 μM and other initial conditions and parameters as listed in Table: default parameter values and initial conditions.

• Figure 5B: Bifurcation plots were calculated as described in subsection ‘Bifurcation diagrams’. PKA was chosen as bifurcation parameter in the range of 0.1 to 0.6 μM, t_*end*_ = 500000 s and 30 iterations were allowed for direct identification of unstable steady states before the algorithm switches to approximative identification. When the unstable steady state was approximated, the first 30 integration steps were ignored in order to prevent a bias resulting from smaller integration steps at the beginning of the simulations.

• Figure 5D: As described in subsection ‘Local sensitivity analysis’.

• Figure 5E: As described in subsection ‘Probing the multi-dimensional parameter space for its influence on bistability’.

• Figure 6A: First, 250 μM [PLN]_*tot*_ (unphosphorylated) were simulated until oligomerization reached equilibrium in the absence of any other species (no inhibitor-1, no phosphatases, no PKA). Second, except for [PKA], all species and parameters were set to their default value as listed in Table: default parameter values and initial conditions. Next, a series of short bursts of [PKA] = 0.59 μM, each 45 s apart, was simulated and burst duration was increased for each simulation (1/3.3/10 s).

• Figure 6B: As in Figure 6A but in absence of inhibitor-1 and with PP1 reduced by the fraction of PP1 bound to inhibitor-1 at steady state from previous simulations (to ensure the same steady state is approached).

• Figure 6C: As in Figure 6A but with rate constants $k_{1}=k_{2}=k_{3}=k_{4}=k_{5}=k_{6}=0$ (to prevent oligomerization) and an initial PLN concentration equimolar to the monomer concentration of [PLN]_*tot*_ = 250 μM at oligomerization equilibrium (≈ 58.2 μM).

• Figure 6D: combined settings of Figures 6B and 6C.

• Figure 6E: First, the model was simulated to steady state with [PKA] = 0.2 μM and other initial conditions and parameters as listed in Table: default parameter values and initial conditions. From the steady state, we simulated the model for 512 short time intervals of 0.1 s length each, where for the i-th time interval, we set [PKA](i) = 0.2 μM + 0.05 ⋅ 0.2 μM ⋅ sin($\frac{\left(i-1\right)}{20}$) rnd()⋅ 0.075 ⋅ 0.2 μM, where the last summand represents a fast random noise component.

• Figure 6F: First, the model was simulated to steady state with [PKA] = 0.2 μM and other initial conditions and parameters as listed in Table: default parameter values and initial conditions. Frequency response analysis for the reached steady state was carried out as described in subsection ‘Frequency response analysis’. For frequency response analysis in the absence of pentamers or inhibitor-1, simulation until steady state was modified as described for Figures 6B and 6C.

• Figure 6G: The parameters of the parameter set yielding ultrasensitivity without bistability which deviate from default conditions are:

**Table:** 

Parameter/IC	Value	Comments
${\left[PP1\right]}_{tot}$	0.67 μM (default: 0.89 μM)	free $\left[PP1\right]\left(t=0\right)={\left[PP1\right]}_{tot}$ unless stated otherwise
$K_{m,PKA:PLN_{1}}$	55 μM (default: 42 μM)	($K_{m}$ for PLN_1_ phosphorylation by PKA)
$K_{m,PKA:PLN_{5}}$	42.5 μM (default: 42 μM)	($K_{m}$ for PLN_5_ phosphorylation by PKA)
$k_{cat,PP1:PLN_{5}}$	9 s^-1^ (default: 8.5 s^-1^)	(baseline $k_{cat}$ for PLN_5_ dephosphorylation by PP1)
$K_{m,PP1:PLN_{5}}$	4 μM (default: 7 μM)	(baseline $K_{m}$ for PLN_5_ dephosphorylation by PP1)
φ	0.79 (default: 0.2)	(v-type cooperativity for PLN_5_ dephosphorylation by PP1)
χ	1.03 (default: 1)	(k-type cooperativity for PLN_5_ dephosphorylation by PP1)

• Bifurcation diagrams were calculated as described for Figure 5B. The response to fluctuations in PKA concentration was determined as follows: First, the model was simulated to steady state with [PKA] = 0.226 μM and other initial conditions and parameters as listed in Table: default parameter values and initial conditions, or as given in the table above for the parameter set leading to ultrasensitivity without bistability. From the steady state, we simulated the model for 100 time intervals of 60 s length each, where for the i-th time interval, we set [PKA](i) = 0.226 μM rnd()⋅ 0.25 ⋅ 0.226 μM.

• Figures 6H and 6I: Noise-landscapes were calculated as follows. First, the model was simulated to steady state at a given baseline PKA concentration [PKA]_*bl*_ and other initial conditions and parameters as listed in Table: default parameter values and initial conditions, or as given in the table above for the parameter set leading to ultrasensitivity without bistability. From the steady state, we simulated the model for 150 time intervals of 60 s length each, where for the i-th time interval, we set [PKA](i) = [PKA_*bl*_] rnd()⋅ $n_{f}$ ⋅ [PKA_*bl*_], where $n_{f}$ is the input noise level. From these simulations, the coefficient of variation (CV = $\frac{\sigma }{\mu }$) of relative PLN monomer phosphorylation was determined, where σ and μ denote the standard deviation and mean of the data, respectively. This process was repeated iteratively and the resulting CVs were visualized on a surface plot as function of the indicated ranges of [PKA]_*bl*_ and input noise levels. For the relative noise landscape shown in Figure 6I, CVs resulting from the parameter set for ultrasensitive without bistability were divided by the CVs resulting from the parameter set for bistability.

• Figure 6J: The model was simulated to steady state at a given baseline PKA concentration [PKA]_*bl*_, where [PKA]_*bl*_ = 0.208 μM for the parameter set exhibiting bistability or [PKA]_*bl*_ = 0.225 μM for the parameter set resulting in ultrasensitivity without bistability. Other initial conditions and parameters as listed in Table: default parameter values and initial conditions, or as given in the table above for the parameter set leading to ultrasensitivity without bistability. From the steady state, we simulated the model for 1000 time intervals of 60 s length each, where for the i-th time interval, we set [PKA](i) = [PKA_*bl*_] rnd()⋅ 0.0625 μM. A switching event between low and high phosphorylation states was counted when relative monomer phosphorylation changed from ≤ 0.25 to ≥ 0.4 or vice versa.

### Quantification and statistical analysis

All image-based experimental data were quantified using the ImageLab v6.0 software (Bio-Rad). Statistical comparison between two experimental groups was performed in GraphPad Prism (v8.3) using the two-tailed unpaired t test, corrected for multiple comparisons by the Holm-Sidak method where appropriate. Sample sizes are given in the respective figures. A p value < 0.05 was considered to be statistically significant. Significance levels were encoded as follows: ns = not significant, ^∗^ p < 0.05, ^∗∗^ p < 0.01, ^∗∗∗^ p < 0.001, ^∗∗∗∗^ p < 0.0001.

## Data Availability

The custom code for models and simulations has been deposited in the BioModels database at: https://www.ebi.ac.uk/biomodels/MODEL2011110001.

## Supplemental information

Document S1. Figures S1–S12 and Table S1

Document S2. Article plus supplemental information
