# Supplementary material for: Molecular noise filtering in the β-adrenergic signaling network by phospholamban pentamers
Source: Cell Rep. 2021 Jul 27;36(4):109448. doi: 10.1016/j.celrep.2021.109448 (PMC8333238; doi:10.1016/j.celrep.2021.109448)
Supplement: Document S1. Figures S1–S12 and Table S1 [file mmc1.pdf]

Cell Reports, Volume *36*

Supplemental information

Molecular noise filtering in the β-adrenergic signaling network by phospholamban pentamers

Daniel Koch, Alexander Alexandrovich, Florian Funk, Ay Lin Kho, Joachim P. Schmitt, and Mathias Gautel

Supplemental Information

Supplemental Figures

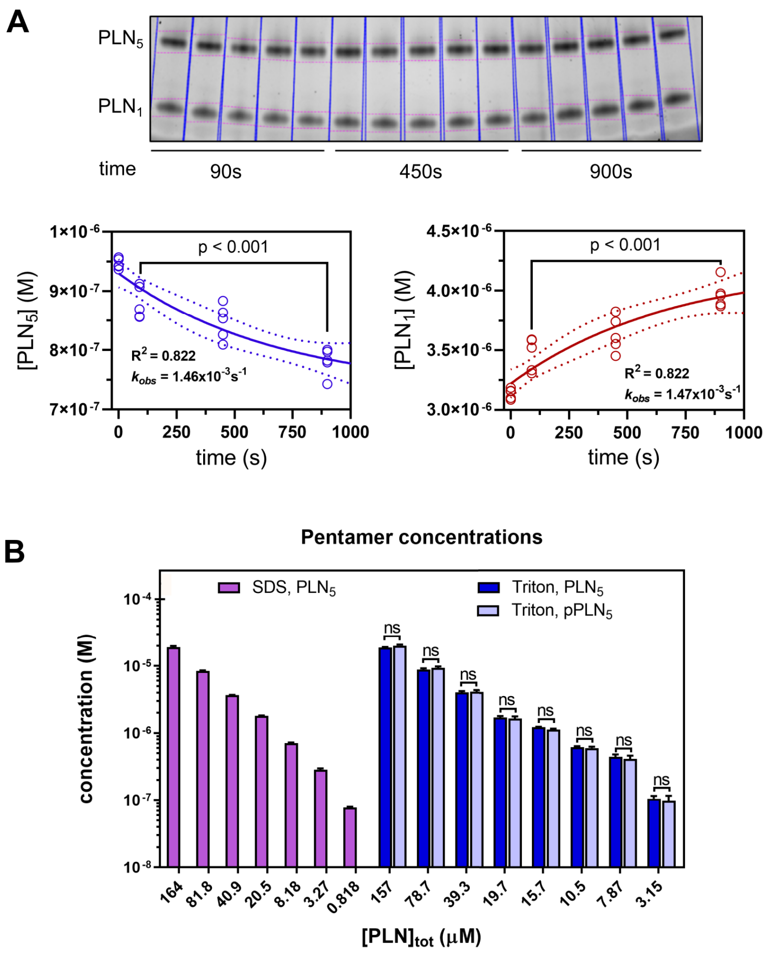

Figure S1

Dissociation dynamics and influence of phosphorylation on pentamerizaton in TBB. (A) PLN dissociation time course. Top: pre-equilibrated PLN at a concentration of ≈ 1 mg/ml was diluted 20-fold and dissociation was allowed to proceed for 90 to 900 s (each experiment was started at different time points to ensure a simultaneous endpoint). Bottom: quantification shows a very slow, but statistically significant ($p < 0.001$) pentameter dissociation and monomer accumulation. Concentrations at $t = 0$ s were calculated from the equilibrium concentration of PLN at 1 mg/ml by division through 20, i.e. assuming no dissociation directly after dilution at $t = 0$ s. **(B)** PLN pentamer concentrations at different total PLN concentrations show no significant differences between unphosphorylated and phosphorylated PLN in TBB (same data as in Figure 1 from the main text). Bars represent mean ±SD.

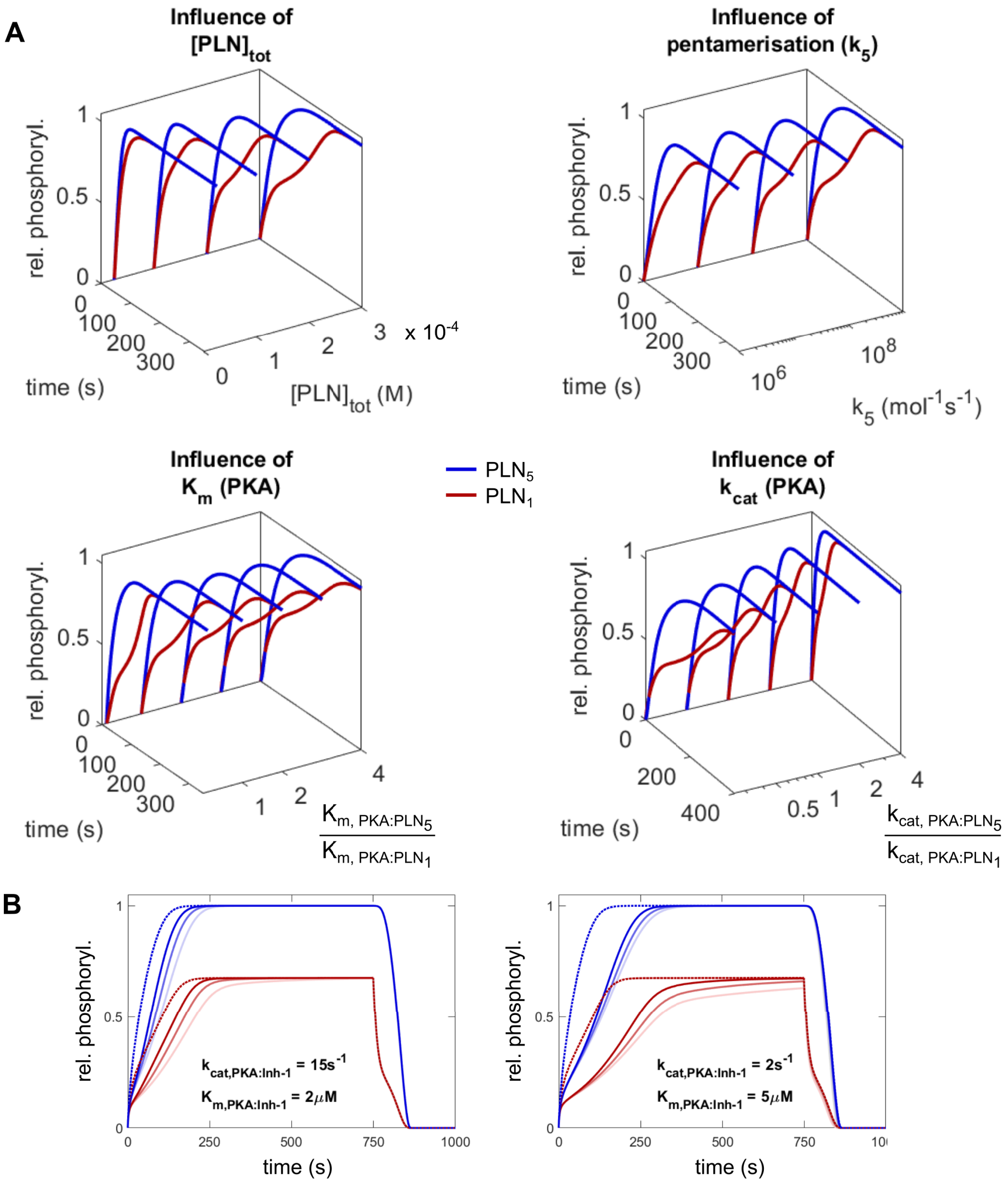

Figure S2

Influence of model parameters on phosphorylation delays due to PLN pentamers and inhibitor-1 FFL. (A) Parameter dependency of monomer phosphorylation delay by PLN pentamers. (Top) Higher $[PLN]_{tot}$ or pentamerization contribute to the monomer phosphorylation delay, likely by increasing the competitive effect of pentamers. (Bottom) Monomer phosphorylation delay is more pronounced if pentamers are a worse substrate for PKA than monomers (i.e. either lower substrate affinity K_m or lower turnover number k_{cat}). Although surprising at first glance, this becomes plausible considering that the competitive effect abates when all pentamers have been phosphorylated. **(B)** Response delays by inhibitor-1 FFL at slower phosphorylation rates of inhibitor-1 by PKA.

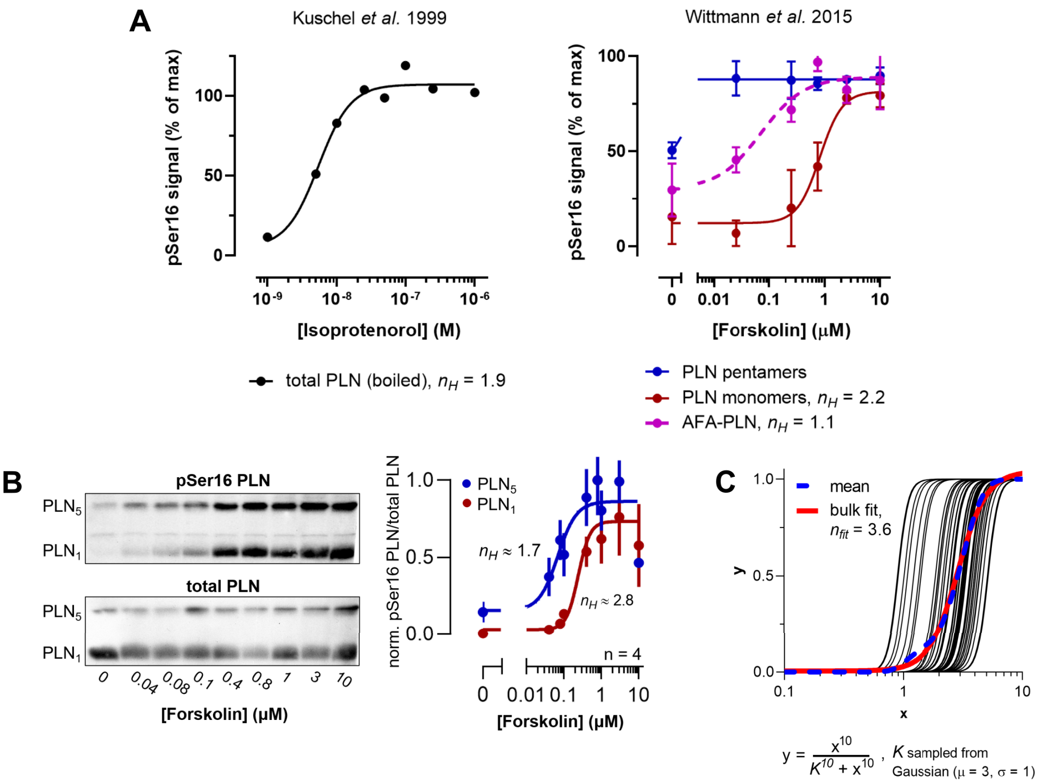

Figure S3

Ultrasensitive PLN phosphorylation. (A) Dose-response data on PLN phosphorylation from the literature was fitted to the Hill-equation with offset. Left: Phosphorylation of total PLN (pentamers were dissociated by boiling) in rat hearts. Right: Differential phosphorylation of PLN monomers and pentamers in transfected and forskolin stimulated HEK293 cells. Note that high Hill-exponents depend on the presence of pentamers. **(B)** Replication of ultrasensitive PLN phosphorylation in transfected and forskolin stimulated HEK293 cells (this study). Data points represent mean ±SEM. **(C)** Although fitted Hill-exponents of experimental data indicate some degree of ultrasensitivity ($n_H \approx 2$), the true response on single cell level could be blunted due to averaging across cells: black lines show 50 Hill-curves generated with $n_H = 10$ and slight variation in the half-saturation constant K. The bulk fit of all curves can lead to reduced Hill-exponents (here: $n_H = 3.6$).

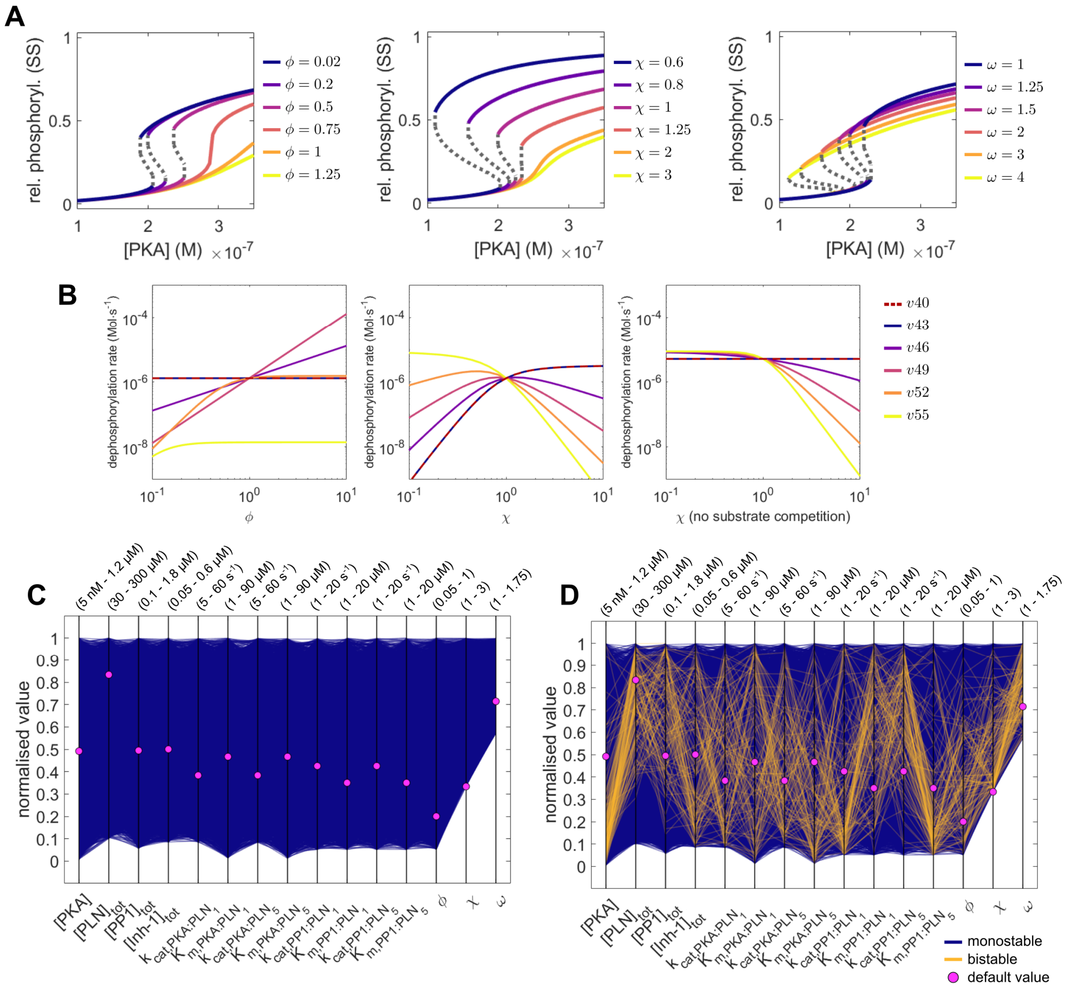

Figure S4

Influence of model parameters on bistability. (A) Bifurcation plots of relative PLN monomer phosphorylation for different strengths and types of PP1 cooperativity (ϕ, χ) and for the dynamic equilibrium of PLN (ω). Parameters, which were not varied, are equal to their default value. **(B)** PP1 dependent dephosphorylation rates for monomeric and pentameric PLN as a function of parameters ϕ and χ. **(C,D)** Random sampling of parameters. **(C)** No bistability could be observed in the absence of pentamers regardless of the values for other kinetic parameters or protein concentrations. **(D)** Implementing k-type cooperativity (parameter χ) reduces the probability for a parameter set to be bistable as only 1.1% of tested parameter sets showed bistability.

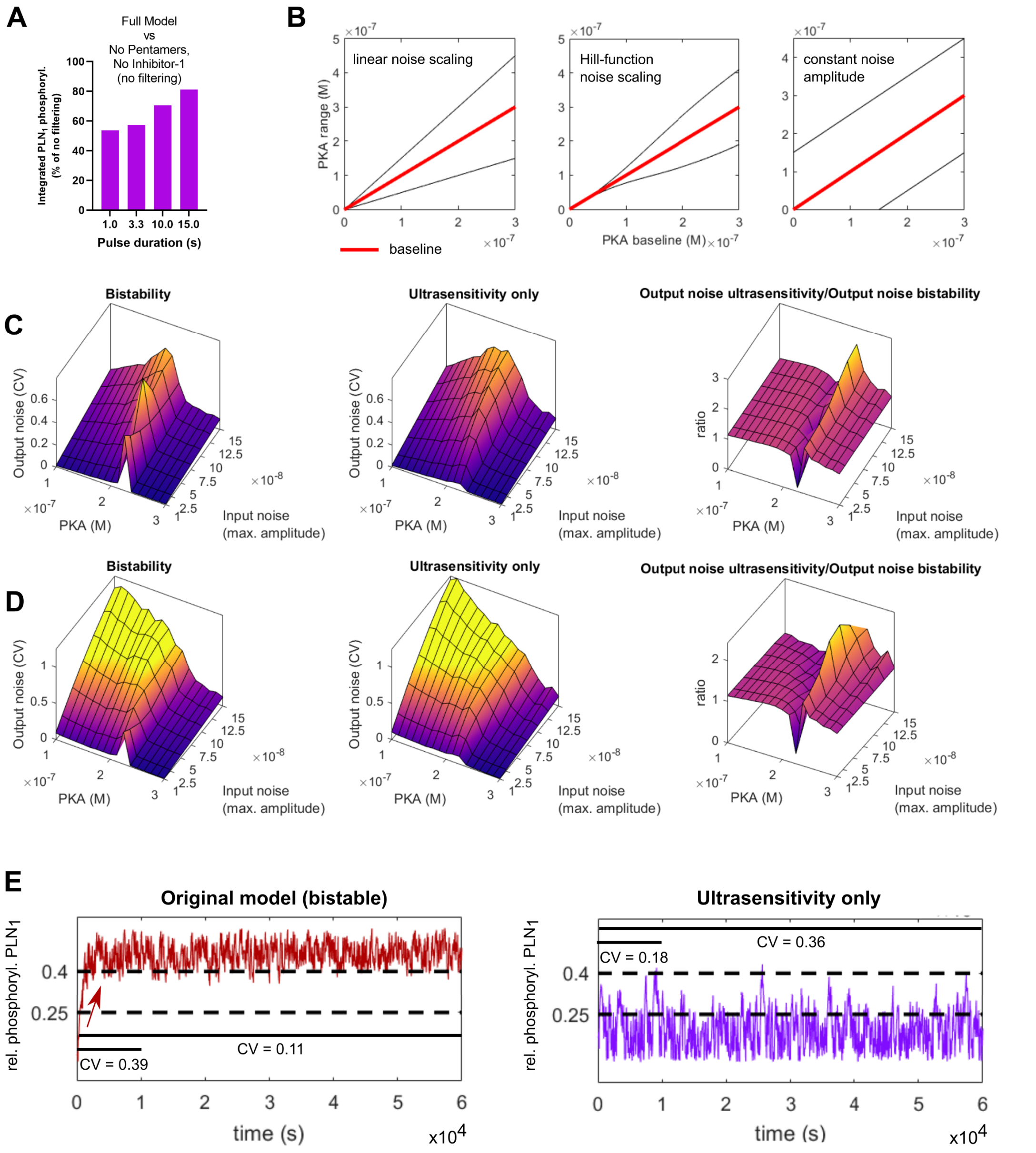

Figure S5

Additional noise-filtering analyses. **(A)** Comparison of the integrated PLN_1 phosphorylation for 4 subsequent bursts/pulses and different pulse durations in the full model vs the model version without pentamers and inhibitor-1. The difference between the model versions becomes smaller for longer pulses. **(B)** Alternative noise functions. Left: linear noise underlying the noise landscapes in the main text. Middle: non-linear noise amplitude as a Hill-function of the baseline [PKA] with $K = 2 \times 10^{-7}$ M and $n_H = 2.5$. Right: constant noise amplitude. **(C)** Absolute and relative noise landscapes with Hill noise. **(D)** Absolute and relative noise landscapes with constant noise amplitude. **(E)** Simulation of 1000 fluctuations at 0.225μM baseline [PKA] and input noise levels of 20% of baseline [PKA] (frequency: 1 min^{-1}). The higher output noise of the bistable model compared to the only ultrasensitive model in short simulations (150 fluctuations) is typically caused by a single switching event (red arrow) and is attenuated in prolonged simulations.

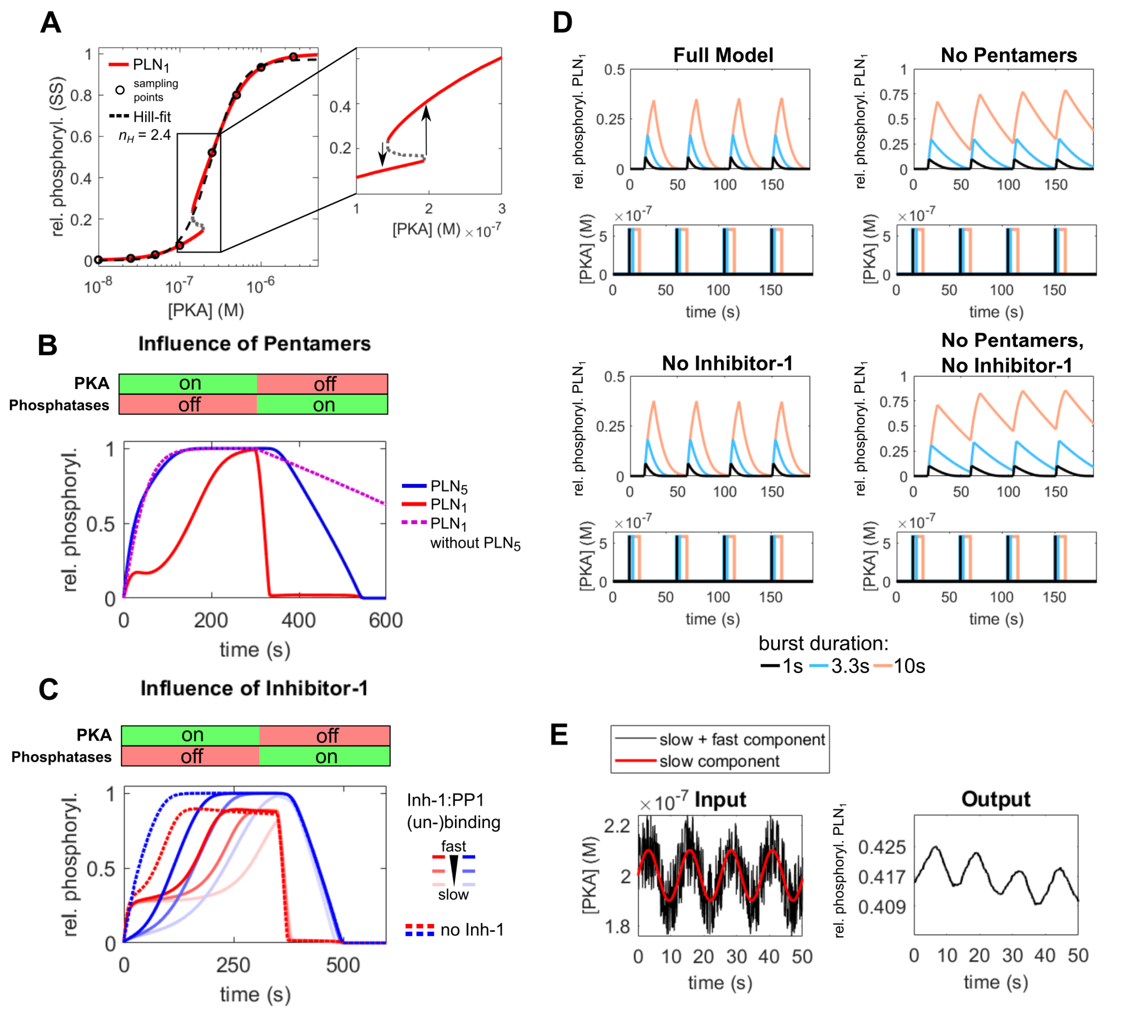

Figure S6

Alternative parameter set with lower ultrasensitivity. **(A)** Sampling the bifurcation diagram at 8 different points leads to a fitted dose-response curve with a Hill-exponent of $n_H = 2.4$ similar to experimental dose-response data. The parameter set still allows for bistability (with even higher bistable range than with the default parameters) and thus is able to filter out small fluctuations close to the critical threshold. **(B,C)** The alternative parameter set leads to stronger phosphorylation delay capacities than the default parameter set. Consequently, short PKA activity bursts are filtered out **(D)** and the PLN network can still act as a low-pass filter **(E)**.

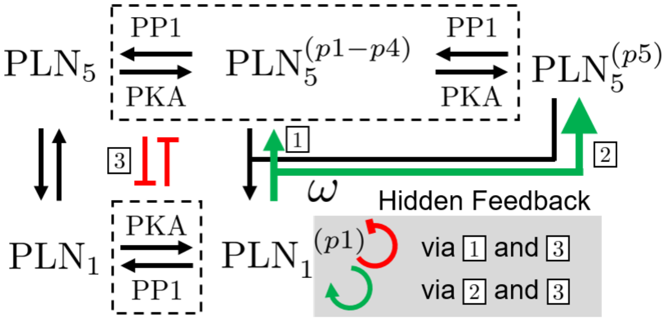

Figure S7

Hidden feedback loops. Since PLN monomers and pentamers compete with each other to be (de-)phosphorylated by PKA and PP1 (3), the dynamic equilibrium could potentially induce hidden feedback loops. First, oligomerization of phosphorylated monomers is in itself a negative auto-loop reducing the amount phosphorylated monomers. If incompletely phosphorylated pentamers are formed (1), monomer phosphorylation could be further decreased due to more pentamers competing with monomers for PKA (3). However, pentamers also compete with monomers for PP1 and could decrease monomer dephosphorylation - particularly if completely phosphorylated pentamers are formed (2). Which of these loops dominates likely depends on the relative reaction velocities among phosphorylation/dephosphorylation steps in a given situation. Hidden feedback loops have been proposed to support the emergence of bistability (see main text for references).

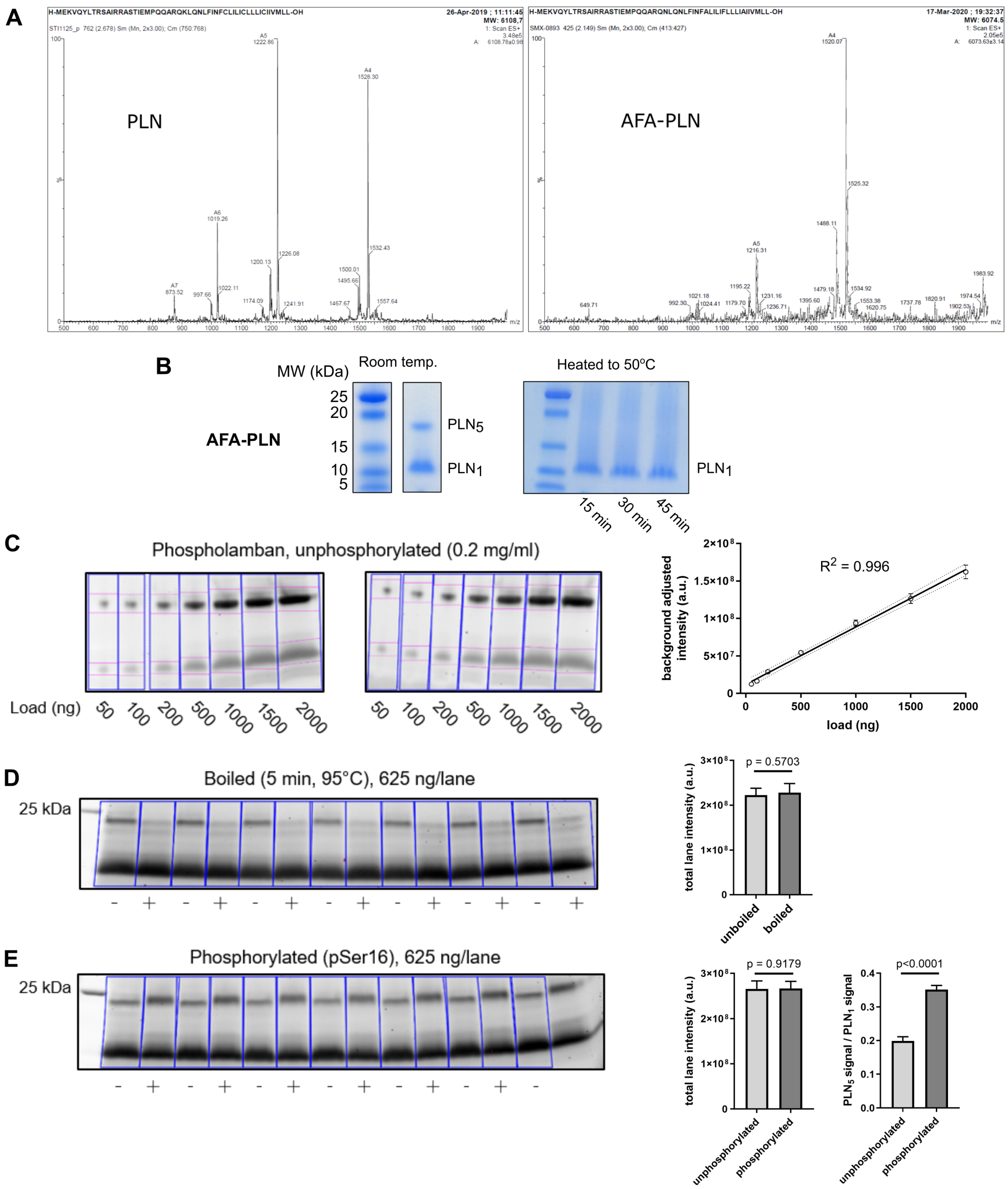

Figure S8

Characterization of synthetic PLN peptides and their quantification via Oriole™ Fluorescent Gel Stain. (A) ES-MS spectra of human wild-type PLN (left) and AFA-PLN (right) show correct molecular weight. **(B)** The Oriole signal of total PLN (monomers + pentamers) in TBB shows excellent linearity for the probed range (50-2000 ng). **(C)** 1 mg/ml of boiled/unboiled PLN in TBB was diluted to 0.05 mg/ml in 1x Laemmli buffer and loaded to the gel. Quantification of total lane intensities suggests that the oligomeric state of PLN does not influence the signal intensity. **(D)** 1 mg/ml of phosphorylated/unphosphorylated PLN in TBB was diluted to 0.05 mg/ml in 1x SDS-sample buffer and loaded to the gel. Quantification of total lane intensities suggests that the phosphorylation state of PLN does not influence the signal intensity. The increased pentamer/monomer signal ratio suggests that Ser16 phosphorylation leads to increased pentamerization in mixed SDS/Triton X-100 micelles. **(E)** SDS-PAGE analysis of the oligomeric status of AFA-PLN at different temperatures and incubation times. 0.33 mg/ml protein concentration, loaded 2 µl per lane. Bars represent mean ±SD.

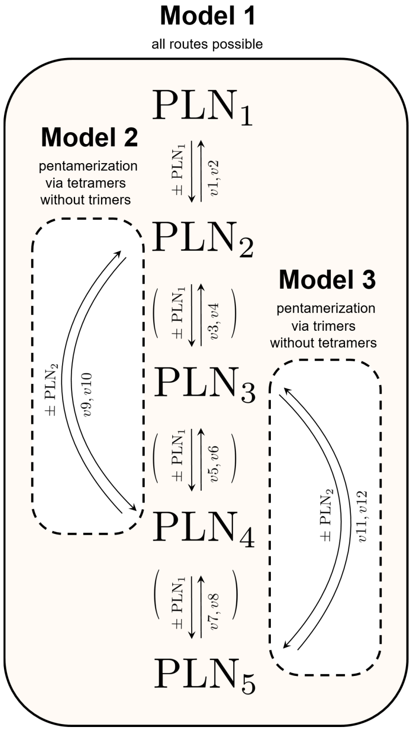

Reaction rates

$v1 = k_1[PLN]^2$
$v2 = k_2[PLN_2]$
$v3 = k_3[PLN_2][PLN]$
$v4 = k_4[PLN_3]$
$v5 = k_5[PLN_3][PLN]$
$v6 = k_6[PLN_4]$
$v7 = k_7[PLN_4][PLN]$
$v8 = k_8[PLN_5]$
$v9 = k_9[PLN_2]^2$
$v10 = k_{10}[PLN_4]$
$v11 = k_{11}[PLN_3][PLN_2]$
$v12 = k_{12}[PLN_5]$

Equilibrium constants

$K_{1,1} = \frac{k2}{k1} = \frac{[PLN_2]}{[PLN][PLN]}$

$K_{1,2} = \frac{k4}{k3} = \frac{[PLN_3]}{[PLN][PLN_2]}$

$K_{1,3} = \frac{k6}{k5} = \frac{[PLN_4]}{[PLN][PLN_3]}$

$K_{1,4} = \frac{k8}{k7} = \frac{[PLN_5]}{[PLN][PLN_4]}$

$K_{2,2} = \frac{k10}{k9} = \frac{[PLN_4]}{[PLN_2][PLN_2]}$

$K_{2,3} = \frac{k12}{k11} = \frac{[PLN_5]}{[PLN_2][PLN_3]}$

ODEs

$$\frac{d}{dt}[PLN_1](t) = 2 \cdot v2 + v4 + v6 + v8 - 2 \cdot v1 - v3 - v5 - v7$$

$$\frac{d}{dt}[PLN_2](t) = v1 + v4 + 2 \cdot v10 + v12 - v2 - v3 - 2 \cdot v9 - v11$$

$$\frac{d}{dt}[PLN_3](t) = v3 + v6 + v12 - v4 - v5 - v11$$

$$\frac{d}{dt}[PLN_4](t) = v5 + v8 + v9 - v6 - v7 - v10$$

$$\frac{d}{dt}[PLN_5](t) = v7 + v11 - v8 - v12$$

$k_3 = k_4 = k_5 = k_6 = k_{11} = k_{12} = 0$ for **Model 2**
$k_5 = k_6 = k_7 = k_8 = k_9 = k_{10} = 0$ for **Model 3**

Figure S9
Mass action kinetics based models of PLN pentamerization. The index denotes different oligomer size, PLN_1 for monomers, PLN_2 for dimers and so forth. Model 1 considers all reaction routes possible, whereas model 2 assumes a monomer→dimer→tetramer→pentamer and model 3 a monomer→dimer→trimer→pentamer pathway.

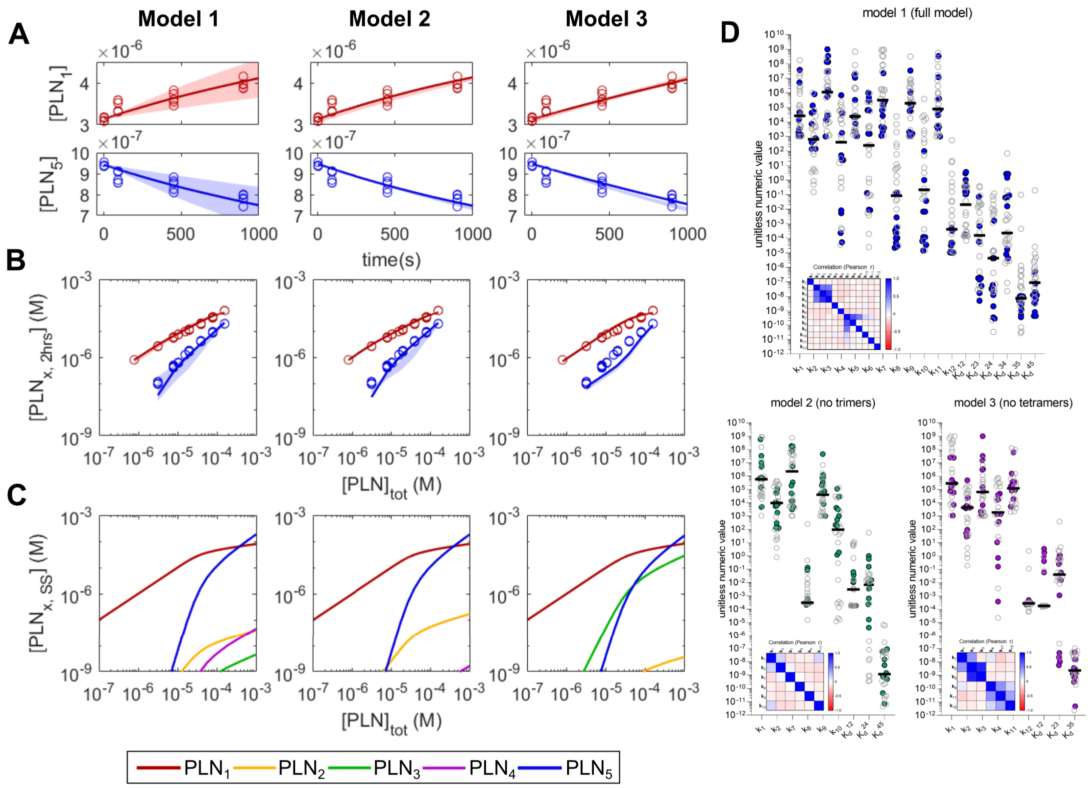

Figure S10

Calibration of the mass action kinetics models. Model fits to experimental dissociation time-course **(A)** and dilution **(B)** data. Continuous lines represent results from the best fit parameter sets, circles are experimental data points, shaded areas represent 95% confidence intervals from the ten best parameter sets. **(C)** Simulated equilibrium concentrations of monomeric and oligomeric PLN at different total PLN concentrations using the best fit parameter set for each model. **(D)** Distribution of fitted parameter values of 30 independent parameter estimation runs for model 1-3. Parameter values associated with the ten best fits are highlighted in colour. Inset shows correlation matrix of the estimated rate constants.

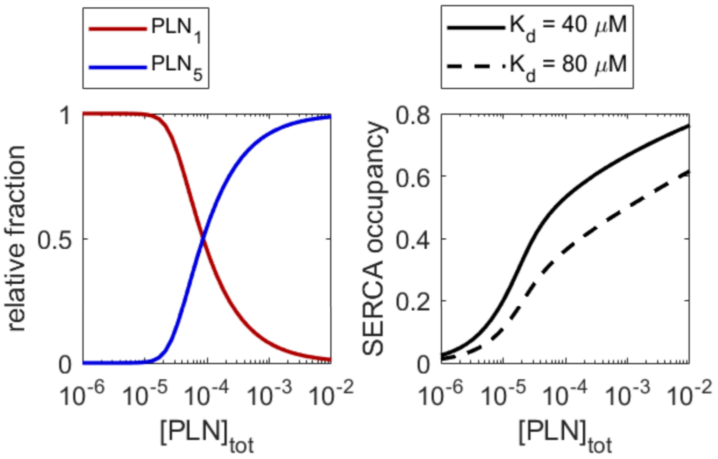

Figure S11

Oligomerization and SERCA occupancy as a function of total PLN concentration. Left: relative fraction of monomers and pentamers as a function of total PLN concentration based on the best fit parameter set for pentamerization model 2. Right: SERCA occupancy by PLN assuming only monomers bind SERCA and that [SERCA] $\ll$ [PLN] so that SERCA binding does not influence pentamerization.

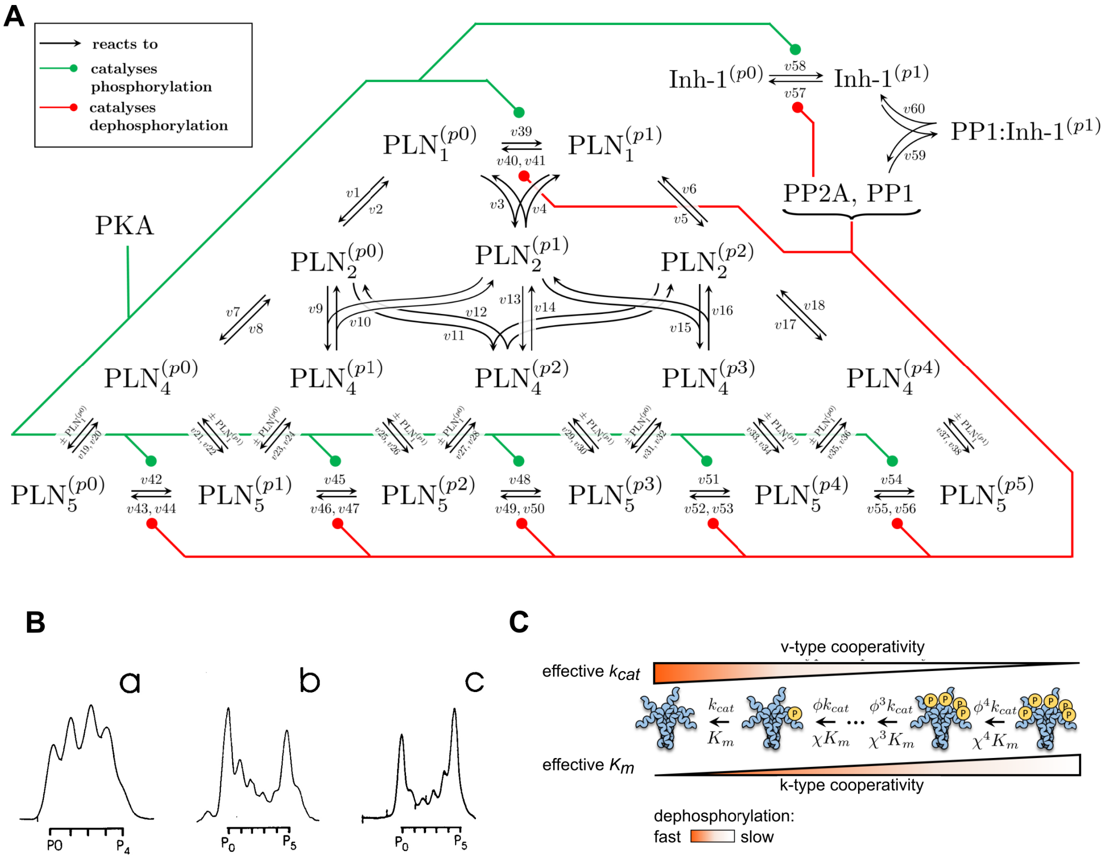

Figure S12

Scheme of the PLN signaling network and kinetic properties of PLN pentamers. (A) Complete reaction scheme of the phospholamban model. **(B)** Distribution of phosphorylated pentamer species indicated non-cooperative and random phosphorylation of pentamers by PKA (a), but strong positive cooperativity for PLN pentamer phosphorylation by CamKII at Thr17 (b) and dephosphorylation of pentamers after PKA phosphorylation (c). Figure reprinted with permission from Colyer, J. (1998). Phosphorylation States of Phospholamban. Ann.N.Y.Acad.Sci. 853, 79-91. **(C)** Illustration of how positive cooperativity of PLN$_5$ dephosphorylation is implemented in the model. Positive cooperativity denotes an enhanced reaction velocity after each dephosphorylation step, which could be a result of faster catalytic turnover (v-type cooperativity, i.e. the value of k_{cat} increases after each step) or higher substrate affinity (k-type cooperativity, i.e. the value of K_m increases after each step). For each phosphate group present in a pentamer which is dephosphorylated by PP1, the effective k_{cat} in the model was thus scaled by a factor $\phi < 1$ and/or the effective K_m was scaled by a factor $\chi > 1$.

Supplemental Tables

Table S1

Qualitative predictions for the expectedinfluence of R14del effects on noise-filtering.

R14del effect	Low-pass filtering	Bistability	Based on
Mistargeting of R14del PLN<br>→ lower $[PLN]_{tot}$ at SR<br>→ lower $[PLN_5]$	↓	↓	sensitivity analysis, Figure S2
destabilization of pentamers<br>→ lower $[PLN_5]$	↓	↓	sensitivity analysis, Figure S2
(wildtype/R14del hetero-pentamers<br>→ fewer phosphorylation sites)	(↓)	(↓)	(Ortega *et al.* 2006)
